# Widespread false gene gains caused by duplication errors in genome assemblies

**DOI:** 10.1186/s13059-022-02764-1

**Published:** 2022-09-27

**Authors:** Byung June Ko, Chul Lee, Juwan Kim, Arang Rhie, Dong Ahn Yoo, Kerstin Howe, Jonathan Wood, Seoae Cho, Samara Brown, Giulio Formenti, Erich D. Jarvis, Heebal Kim

**Affiliations:** 1 Department of Agricultural Biotechnology and Research Institute of Agriculture and Life Sciences, Seoul National University, Seoul, Republic of Korea; 2 Interdisciplinary Program in Bioinformatics, Seoul National University, Seoul, Republic of Korea; 3 Genome Informatics Section, Computational and Statistical Genomics Branch, National Human Genome Research Institute, National Institutes of Health, Bethesda, USA; 4 Wellcome Sanger Institute, Cambridge, UK; 5 eGnome, Inc, Seoul, Republic of Korea; 6 Laboratory of the Neurogenetics of Language, The Rockefeller University, New York, NY USA; 7 Howard Hughes Medical Institute, Chevy Chase, MD USA

**Keywords:** False duplication, Assembly error, Phasing error, De novo assembly, Vertebrate genome project

## Abstract

**Background:**

False duplications in genome assemblies lead to false biological conclusions. We quantified false duplications in popularly used previous genome assemblies for platypus, zebra finch, and Anna’s Hummingbird, and their new counterparts of the same species generated by the Vertebrate Genomes Project, of which the Vertebrate Genomes Project pipeline attempted to eliminate false duplications through haplotype phasing and purging. These assemblies are among the first generated by the Vertebrate Genomes Project where there was a prior chromosomal level reference assembly to compare with.

**Results:**

Whole genome alignments revealed that 4 to 16% of the sequences are falsely duplicated in the previous assemblies, impacting hundreds to thousands of genes. These lead to overestimated gene family expansions. The main source of the false duplications is heterotype duplications, where the haplotype sequences were relatively more divergent than other parts of the genome leading the assembly algorithms to classify them as separate genes or genomic regions. A minor source is sequencing errors. Ancient ATP nucleotide binding gene families have a higher prevalence of false duplications compared to other gene families. Although present in a smaller proportion, we observe false duplications remaining in the Vertebrate Genomes Project assemblies that can be identified and purged.

**Conclusions:**

This study highlights the need for more advanced assembly methods that better separate haplotypes and sequence errors, and the need for cautious analyses on gene gains.

**Supplementary Information:**

The online version contains supplementary material available at 10.1186/s13059-022-02764-1.

## Background

Biological misinterpretations can occur when genomic regions unknowingly have errors. But it is unclear as to the magnitude of mis-assembly errors in existing genome assemblies, generated in the transition from the fragmented DNA sequences to the assembled blueprint of a species [1–8]. Followed by the first assembly of fruit fly in 2000 [9] and a human reference genome in 2003 [10], ~100 reference genomes of vertebrates were deposited in public databases by 2010 using mostly intermediate read length (~700 bp) Sanger reads. The number of genomes gradually increased to ~700 by 2018, mostly using short read-based (~35–250 bp) next generation sequencing (NGS) [11]. These genomes helped bring about discoveries in a variety of fields, including evolution, ecology, agriculture, and medicine [12–17]. However, with short read-based assemblies, it was difficult to resolve repeat regions longer than the read lengths [1, 18–20].

Preliminary studies have indicated that the longer the sequence read length, the less likely an assembly structural error [1], which has been quantitatively validated in our companion Vertebrate Genomes Project (VGP) flagship study in 2021 [5]. An underappreciated source of mis-assembly was heterozygosity [5, 21]. Mis-assignment of heterozygous genomic regions led to both copies of the partnering alleles being assembled as paralogs in the same haploid assembly [3, 5, 6], which are called false heterotype duplications by the VGP [5]. Likewise, accumulated sequence errors in reads, particularly long reads, led to under-collapsed sequences, which were called homotype false duplications [5]. Both heterotype and homotype false duplications in genic regions can be misinterpreted as gene gains [1, 21, 22]. The VGP proposed that these false gains happen in more highly divergent regions of the genome, where assembly algorithms have difficulty distinguishing haplotype homologs from haplotype paralogs [5], but this was not quantitatively tested in regards to the type of duplication.

Although long-read sequencing is better at resolving repetitive regions [1, 11, 23], they alone are unable to fully resolve false duplications [1, 24, 25]. One way to prevent false duplications is to make homozygous lineages through inbreeding. But this can be either impossible or very difficult under most circumstances [25, 26], especially if one were to sequence all species of a lineage, such as the goal of the VGP that aims to produce complete and error-free reference genomes for all ~70,000 vertebrate species [27–29]. Another way to solve false duplications is to use assembly strategies for efficient haplotype phasing, some developed and applied in the VGP [5, 25, 30, 31]. But most of the non-VGP vertebrate genomes in the public databases as of to date have been reconstructed without haplotype phasing. A full quantitative and qualitative assessment has not been conducted on the prior versus VGP genomes to determine the extent and types of false duplications, and improvements in the VGP assemblies.

Here we performed a detailed analysis to measure the presence, magnitude, and cause for false duplications in previous common reference assemblies and their VGP counterparts. We focused on three species, the platypus and zebra finch that were originally assembled using Sanger reads published in 2008 [32] and 2010 [33], respectively, and the Anna’s hummingbird that used short Illumina reads published in 2014 [12, 34]. These are popular references, with the associated studies collectively cited over 3600 times as of April 2021 (Google Scholar). The VGP version of the assemblies were long-read based and used algorithms to phase haplotypes and purge false duplications at multiple steps in the assembly pipeline. We found widespread false duplications in previous assemblies that were corrected in the VGP assemblies, and also identified areas for improvement in current and future assemblies.

## Results

### Genome assemblies and identifying false duplications

The previous Sanger-based platypus [32] and zebra finch [33] reference genomes used standard pipelines for the best reference chromosomal level genomes at the time, generated with 500–1000 bp Sanger sequence reads, BAC-based scaffolding and FISH or cytogenetic chromosome mapping and assignments. No systematic effort was made for haplotype phasing, but both the previous zebra finch and platypus assemblies were rigorously manually curated. The prior Illumina-based Anna’s hummingbird reference [12, 34] was generated with short reads (~150 bp), and contigging and scaffolding with multiple paired-end and mate-pair libraries ranging from 200 bp to 20 kbp in size. An effort was made to remove alternate haplotypes during assembly.

The VGP assemblies of the same species was generated with PacBio-based continuous long-read (CLR) contigs (N50 read length ~17 kbp), which were scaffolded with 10X Genomics linked reads, Bionano Genomics optical maps, and Arima Genomics Hi-C chromatin interaction read pairs [5]. Systematic attempts to prevent false duplications were made, using FALCON-Unzip to separate haplotypes after generation of contigs and purge_haplotigs [35] that search for and purged false heterotype duplications from the primary pseudo-haplotype assembly [5]. All VGP assemblies were subjected to rigorous manual curation to minimize assembly errors generated by algorithmic shortcomings. The previous and VGP assemblies of the zebra finch and Anna’s hummingbird were conducted on genomic DNA from the same individuals, and thus, differences would only be due to sequencing platform and assembly methods. As the platypus was from a different individual, we performed some additional steps later in the study to validate whether the issues found were due to sequence and assembly errors, and not individual biological differences.

The sizes of the previous assemblies of the zebra finch, hummingbird, and platypus are 1.23 Gbp, 1.11 Gbp, and 2.00 Gbp, respectively (Additional file 1: Table S1). They consisted of a total of 37,421, 54,736, and 958,970 scaffolds. Among the scaffolds, 35 and 19 super scaffolds were assigned to chromosomes for the zebra finch and platypus assemblies, respectively. The assemblies had 87,710, 70,084, and 243,835 gaps, and their average contig NG50s were 47.9, 27.0, and 11.4 kbp, respectively. The size of the VGP assemblies were all 0.05–0.17 Gbp smaller (Additional file 1: Table S1). They consisted of ~280- to 3140-fold fewer scaffolds (i.e., 135, 159, and 305 total), of which 33 (now 39 in our updated version), 33, and 31, respectively, were assigned to chromosomes, including the sex chromosomes. The number of gaps likewise were ~160- to 470-fold lower, and contig NG50s were ~250- to 1320-fold higher: 12.0, 13.4, and 15.0 Mbp for the zebra finch, hummingbird, and platypus, respectively. Alternate haplotype scaffolds of 0.95–1.58 Gbp in total size were separated from the primary assembly.

**Fig. 1:**
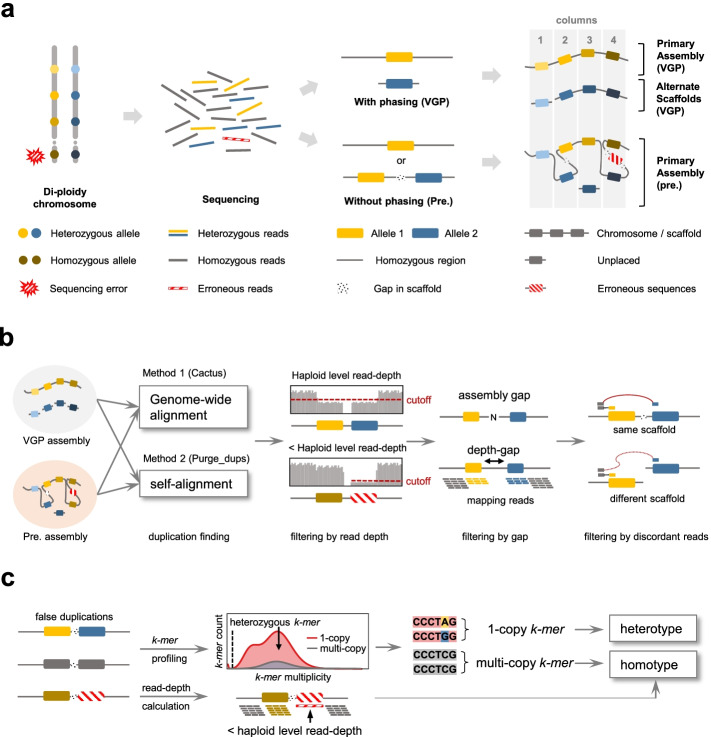
Overview to identify false duplication. **a** Mechanisms of how false assembly duplications are created. If haplotype phasing is included and correctly performed in the assembly process, there will be only one allele in the primary assembly, with the other placed in the alternate assembly (right panel, column 1). However, without proper phasing, both alleles of heterozygous loci may be assembled in one scaffold (column 2) or two different scaffolds (column 3) of the primary assembly. Alternatively, randomly or systematically piled up erroneous sequencing reads can generate false duplications (column 4). This leads to three types of false duplications. **b** Scheme to identify false duplications. Whole-genome alignment between the two assemblies using Cactus and self-alignment using purge_dups reveal candidate false duplicated regions or whole contigs. The union-set from these two independent methods is then used to find false duplications, which contain some combination of near haploid read-depth of the 10X Genomics linked reads, the presence of gaps between duplications, and discordance in read pairs between duplications. **c** Scheme to classify false duplication types. Copy number and multiplicity of *k-mers* are calculated from the assembly and the 10X Genomics linked reads respectively and used to classify false duplications as heterotype or homotype. Heterotype duplication includes haploid specific *k-mers* (i.e., 1-copy). Homotype duplication does not include haploid specific *k-mers*, but does include sequencing errors that can be detected by read-depth below the haploid-level

We performed self-alignment of each assembly using Minimap2 [36] as a part of the purge_dups [31] process to detect duplications independently from another assembly; purge_dups was created by members of the VGP in order to identify and purge false duplications in different contigs. Also, we aligned the previous assemblies to the new VGP assemblies of each species using the reference-free Cactus aligner [37], which allows pair-wise detection of duplicates between the previous and new assemblies at the sequence and contig levels (Fig. 1a, b). We distinguished false duplications from true duplications, as we found that the former had read coverage at the haploid-level, gaps between duplications due to mis-assembly, and discordance in 10X linked read pairs mapped back to the assembly. We classified each false duplication as heterotype duplications when heterozygous *k-mers* were found, and homotype duplications when read depth coverage was lower than the haploid-level, which occurs with sequence read errors, or when heterozygous *k-mers* were not found (Fig. 1c).

### False duplications in previous and VGP assemblies

**Fig. 2:**
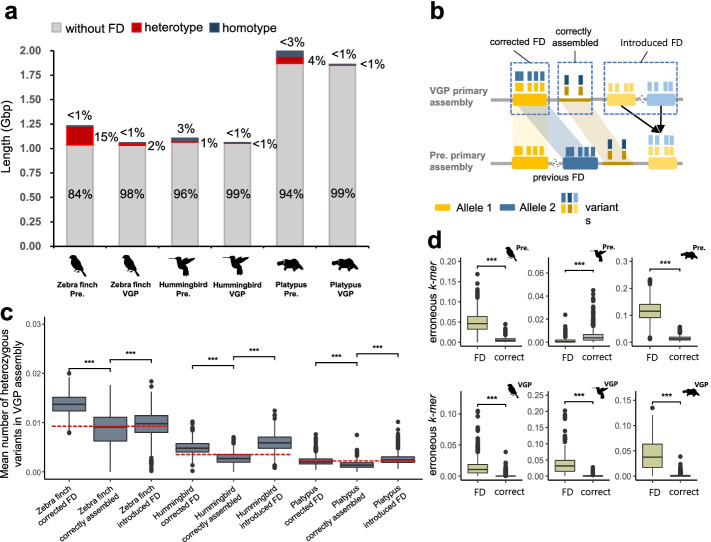
The amount of false duplication and factors that correlate with false duplication. **a** The total assembly size and the proportion that are false duplications in the previous and VGP assemblies. False duplications were classified as heterotype and homotype. **b** Scheme of false duplications (FD) in the previous and VGP assemblies due to heterozygous alleles. Corrected FD are regions in the VGP assembly that are false duplications in the previous assembly. Correctly assembled are regions without any false duplication in the previous and VGP assemblies. Introduced FD are false duplications introduced in the VGP assembly that were not present in the previous assembly. **c** Heterozygosity of corrected FD, correctly assembled, and introduced FD, according to the VGP assembly haplotype data (***P < 0.001; two-sided *T*-test). Red dotted line, overall heterozygosity of the genome. **d** The portion of erroneous *k-mers* in false duplications and correct regions of each assembly (***P < 0.001; two-sided *T*-test)

The distributions of 10X Genomic linked read depth coverage (Additional file 2: Fig. S1) and *k-mer* multiplicity (Additional file 2: Fig. S2) showed that previous assemblies included significant amounts of false duplications: 16% (196 Mbp), 4% (41 Mbp), and 6% (126 Mbp) of the total length of the prior zebra finch, Anna’s hummingbird, and platypus assemblies, respectively (Fig. 2a, Table 1). As the 10X Genomics linked reads were generated on the new platypus individual used, we also found the Sanger raw reads generated from the prior individual in the NCBI Trace Archive and found 104 Mbp of haploid coverage lower than the genome-wide average indicating that the vast majority of the 126 Mbp found with the 10X linked reads are not due to individual differences, but false duplications. This is a whole chromosome’s worth of false duplication (6% of the genome), and thus also unlikely due to individual differences. The higher levels of false duplication found with the 10X linked reads could be due to its 10-fold higher sequence coverage (60X) relative to the Sanger read coverage (6X). For all three previous assemblies, heterotype was the major source of false duplication, an order of magnitude higher than the homotype except for the previous Anna’s hummingbird assembly (Fig. 2a, Table 1). Of the total false duplications, 7 to 24% were on the same scaffold (Table 1).

**Table 1:** False duplication statistics in previous and VGP assemblies

		Zebra finch Pre. (Sanger)	Zebra finch VGP (bTaeGut1)	Hummingbird Pre. (Illumina)	Hummingbird VGP (bCalAnn1)	Platypus Pre. (Sanger)	Platypus VGP (mOrnAna1)
**Heterozygosity (%)**		-	0.95	-	0.34	-	0.22
**Total false duplication length (Mbp)**		195.6 (15.9 %)	24.1 (2.3 %)	40.9 (3.7 %)	5.8 (0.5 %)	126.0 (6.3 %)	5.6 (0.3 %)
**Type**	**Heterotype (Mbp)**	190.5 (15.5 %)	23.1 (2.2 %)	13.7 (1.2 %)	5.4 (0.5 %)	72.0 (3.6 %)	5.0 (0.3 %)
	**Homotype (Mbp)**	5.1 (0.4 %)	0.9 (0.1 %)	27.2 (2.5 %)	0.4 (<0.1 %)	54.0 (2.7 %)	0.6 (<0.1 %)
**Location**	**Same scaffold (Mbp)**	46.4 (3.8 %)	22.2 (2.1 %)	2.9 (0.3 %)	4.8 (0.5 %)	22.7 (1.1 %)	3.4 (0.2 %)
	**Different scaffold (Mbp)**	149.3 (12.1 %)	1.9 (0.2 %)	38.0 (3.4 %)	1.0 (<0.1 %)	103.3 (5.2 %)	2.2 (0.1 %)
**Total assembly Length (Mbp)**		1232.1 (100 %)	1058.0 (100 %)	1105.7 (100 %)	1059.7 (100 %)	1995.6 (100 %)	1858.5 (100 %)

Heterozygosity (top row) was calculated as the mean number of variants in each assembly based on the 10X linked reads produced for each VGP assembly. Second row is the total Mbp (and % of genome size in brackets) that are falsely duplicated. Rows below that are the type and location of false duplications

False duplications in the VGP assemblies were still present, but 7- to 22-fold less: 2.3% (24 Mbp), 0.5% (5.8 Mbp), and 0.3% (5.6 Mbp) of the total primary assembly in the zebra finch, hummingbird, and platypus, respectively (Fig. 2a, Table 1). Heterotype was also the major type of false duplication. In contrast to the prior assemblies, there was a much higher proportion of the false duplications, 61–92%, found on the same scaffold in the VGP assemblies, due to improved scaffolding using multiple long-range sequencing platforms.

### High heterozygosity and sequencing errors associated with false duplications

**Fig. 3:**
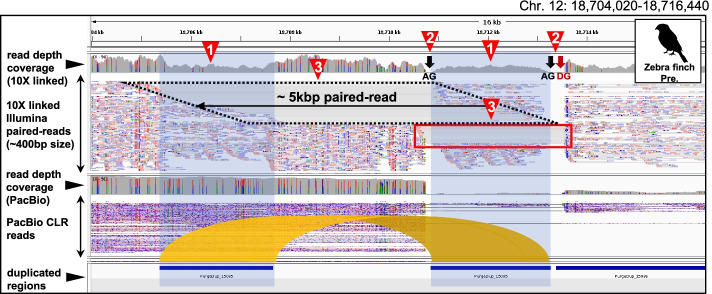
The presence of a gap and discordant reads between false duplications. Shown is a locus in the previous zebra finch assembly with a false duplication. 10X linked read alignments are shown above the PacBio CLR read alignments, along with the depth coverage of the respective read data. Characteristics of false duplications are marked with red triangles: (1) Nearly half depth-coverage and lack of heterozygous variants—colors indicating nucleotide heterozygosity; (2) gaps between false duplications; and (3) discordant 10X linked reads (black dotted box). Red box, discordant reads found near the end of scaffolds that should be connected to each other. AG, assembly gap. DG, depth-gap (unsupported sequences by reads; see the “ Materials and methods” section)

The heterozygosity of false duplications found in the previous assemblies that were corrected in the VGP assemblies (Corrected FD regions; Fig. 2b) were all ~1.5 to 1.8-fold higher than correctly assembled regions without false duplications in both the previous and VGP assemblies (*P* < 0.001; Fig. 2c). The heterozygosity of false duplications specific to the VGP assembly (Introduced FD regions; Fig. 2b) were all also higher (*P* < 0.001) with no specific absolute level that differed with the previous assemblies (Fig. 2c). We also found more erroneous *k-mers* in false duplications than in the correctly assembled regions in both the previous and VGP assemblies (Fig. 2d). Further, regions between the false duplications were most often separated by an assembly gap and sometimes connected by unsupported sequence read depth gaps, due to incorrect gap filling or other assembly errors (Fig. 3; Additional file 2: Fig. S3a,b,c). These properties were not found for true duplications, including of the acrosin (*ACR*) gene and an allele-specific tandem duplication we found in the same contig with haploid level read depth coverage (Additional file 2: Fig. S4a,b). These findings show that increased relative heterozygosity, especially those at the boundaries of homozygous and heterozygous sites, and sequencing errors are prone to be falsely duplicated.

### False duplications cause false annotation errors

**Fig. 4:**
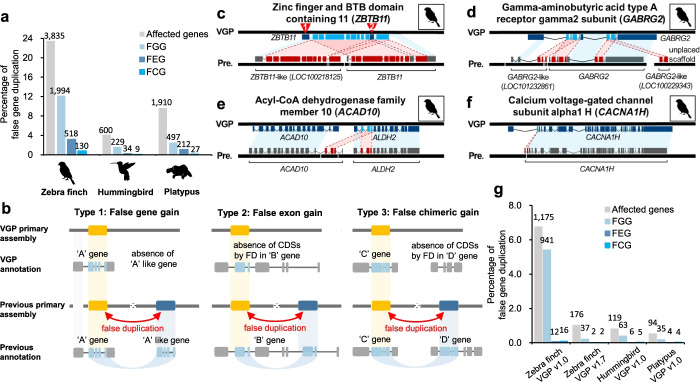
Mis-annotations due to false duplications. **a** Amount and percentage of all genes with mis-annotations caused by false duplications in the previous assemblies. The amount of genes of each type is shown on the top of each bar graph. **b** Types of mis-annotations caused by false duplications. When >50% of the CDS length of a gene was falsely duplicated and annotated as another gene, and resulted in two genes with similar function (e.g., -like), we classified it to false gene gain (FGG, Type 1). When an exon within a gene was falsely duplicated, we classified it to false exon gain (FEG, Type 2). If the duplicated exon was falsely inserted to another existing gene of different function, we classified it as a false chimeric gain (FCG, Type 3). **c** FGG of *ZBTB11*. **d** FGG of *GABRG2*. **e** FCG involving *ACAD10* and *ALDH2*. **f** FEG within *CACNA1H*. The red lines represent the connection between false duplications and the homologs in the VGP assembly. The blue boxes represent the homologous region between the VGP and previous assemblies. The white spaces in the black bars represent scaffold assembly gaps. **g** Amount of false gene annotation in VGP assemblies. The zebra finch VGP v1.0 assembly had false duplications purged with purge_haplotigs after scaffolding; the zebra finch VGP v1.7 had false duplications purged with purge_dups before scaffolding

Among the false duplications, we found 4 to 24% of the coding genes were impacted in the previous assemblies, depending on species (Fig. 4a). Of these, we found three main types: (1) the majority being false gene gains [FGG] of nearly the entire coding sequence; (2) followed by false exon gains [FEG] within a gene; and (3) a minority being false chimeric gains [FCG] from a chimeric join among exons from different genes (Fig. 4a, b; Additional file 3: Table S2).

**Fig. 5:**
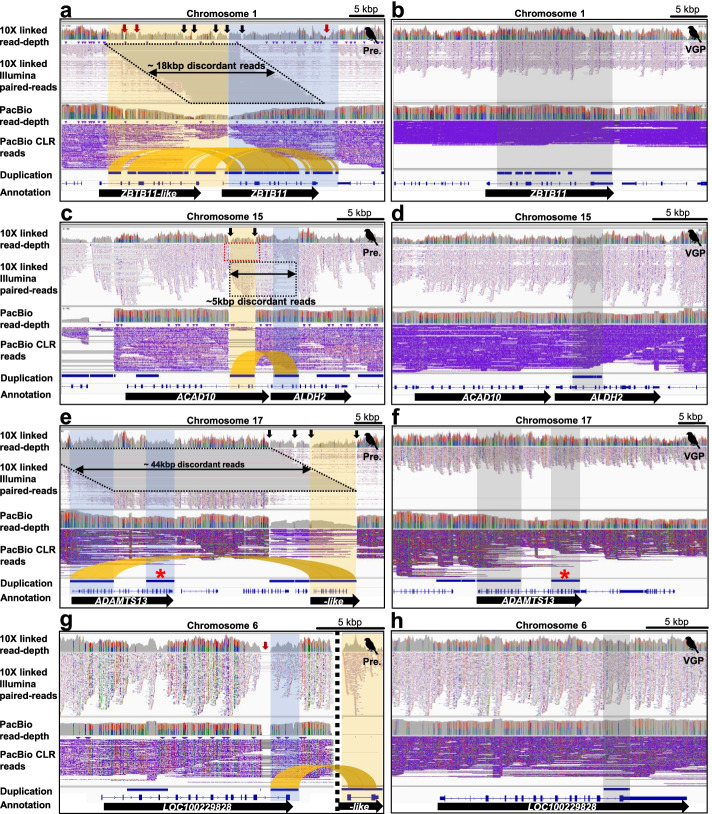
The genome landscape of false gene gains. 10X linked read pairs are shown above the PacBio CLR reads along with the depth coverage of the respective read data. **a** The genome landscape of *ZBTB11* and false *ZBTB11*-like (*LOC100218125*) genes in the previous zebra finch assembly. Most of the region of *ZBTB11* was duplicated adjacent to itself in the previous assembly (highlighted as orange and blue) and showed typical characteristics of false heterotype duplications. Black and red arrows represent assembly gap and read depth-gap, respectively. **b** Corrected gene structure in the VGP assembly (gray). **c** The genome landscape of *ACAD10* and *ALDH2* genes in the previous zebra finch assembly. Three exons of *ALDH2* were inserted in *ACAD10* by a false duplication (highlighted as orange and blue). Black arrows represent assembly gaps. **d** Corrected gene structure in the VGP assembly. The extrinsic three exons from *ALDH2* (gray) were not found in *ACAD10* of the VGP assembly. **e** The genome landscape of *ADAMTS13* and *ADAMTS*-like (*LOC105760960*) genes in the previous zebra finch assembly. The 5′ and 3′ exons of *ADAMTS13* (highlighted as blue) were falsely duplicated to two *ADAMTS13*-like genes, which are assembled in the same scaffold (highlighted as orange) as *ADAMTS13*-like (*LOC105760960*) and a different scaffold (*LOC101232819*; Additional file 2: Fig. S7). The homologous region of the *LOC101232819* gene is marked by a red star. **f** Corrected gene structure in the VGP assembly (gray). The homologous region of the *LOC101232819* gene in the previous assembly is marked by a red star. **g** The genome landscape of neurotrypsin (*LOC100229828*) and neurotrypsin-like genes (*LOC100217566*) in the previous zebra finch assembly. The 3′ region of *LOC100229828* (highlighted as blue) was falsely duplicated to a different small scaffold (~3 kbp; highlighted as orange), NW_002201465. **h** Corrected region in the VGP assembly (highlighted as gray). The different colors and their heights in the read depth rows are the proportion of sites in reads with haplotype variants

An example of a FGG included *ZBTB11* in the previous zebra finch assembly, which had 9 of the 11 coding exons falsely duplicated and annotated as *ZBTB11*-like (*LOC100218125*; Fig. 4c). The non-duplicated *ZBTB11* exon 1 was included in *ZBTB11*-like and exon 2 (red mark; 10th exon) included in *ZBTB11*, while these exons were assembled into one gene in the VGP assembly. The sequence alignment landscape of *ZBTB11* in the previous assembly showed typical characteristics of a false gene gain (Fig. 5a), whereas there was no sign of false duplications in the VGP assembly (Fig. 5b). The gamma-aminobutyric acid receptor subunit gamma 2 (*GABRG2*) was a complex example, where several false exon duplications were assembled in the same scaffold and annotated as a *GABRG2*-like (*LOC101232861*) or as another *GABRG2*-like (*LOC100229343*) on another scaffold, both with presumed false exon losses after the duplication from the original gene (Fig. 4d). Because true duplications can also be annotated as gene name-like, for example *ACR* and *ACR*-like (Additional file 2: Fig. S4a), the “like” term in the NCBI annotation cannot be taken alone as evidence of a false duplication.

*ALDH2*, a gene with specialized upregulation in the zebra finch vocal learning nucleus HVC [38], had three false duplicated exons that were incorporated into the adjacent *ACAD10* gene, causing a FCG for *ALDH2-ACAD10*, all with gaps around each of the false duplications (Figs. 4e and 5c), none present in the VGP assembly (Fig. 5d). The calcium voltage-gated channel subunit alpha1 H (*CACNA1H*), also a gene with specialized expression in vocal learning circuits of the zebra finch [39, 40], had a FEG in the second exon (Fig.4f). Similar examples of FGG, FCG, and FEG in the previous Anna’s hummingbird and platypus assemblies are shown in Additional file 2: Fig. S5. This includes false duplications that overlap in the CDSs of *ATF3*, *PCBD1*, and *VAMP4* in the previous hummingbird assembly and of *ZP2*, *UPF2*, and *HSF2* in the previous platypus assembly with haploid-level Sanger read coverage (Additional file 2: Fig. S6).

We next scanned the literature for reported cases of gene duplications in one or more of the three species studied here and assessed whether they were real or false duplications. There were many cases where the gene duplications were real, but also multiple cases where they were false. An example of the latter included *ADAMTS13,* related to thrombotic thrombocytopenic purpura in humans [41, 42], which was reported as duplicated in the zebra finch [42]. But we found that two of the three *ADAMTS13* genes (one *ADAMTS13* and two *ADAMTS13*-like) were falsely duplicated in the same (*LOC105760960*; Fig. 5e) and a different scaffold (*LOC101232819*; Additional file 2: Fig. S7), respectively. These two false duplications were produced from the 5′ and 3′ ends of the original *ADAMTS13* gene (Fig. 5f). We confirmed that there were no additional copies of *ADAMTS13* in both the VGP zebra finch assembly and a recent VGP chicken assembly (GRCg7w; Accession# GCA_016700215.2). Another example was neurotrypsin, a gene known to be linked to neural development, which was represented as having more copies in the zebra finch than chicken [33]. But we found that this extra copy of the gene (*LOC100217566* in a short unplaced scaffold ~3 kbp long) was made by a false duplication the original neurotrypsin gene in chromosome 6 (*LOC100229828*; Fig. 5g, h), which is annotated as *NTL* in GRCg7w. This extra copy of the gene was not found in both the VGP zebra finch and GRCg7w assemblies. A third example was a platypus vomeronasal receptors (V1R) gene family expansion reported as a sensory adaptation for underwater life history [32]; we found that 43 of the 267 annotated V1R genes (16%) are actually false duplications in the previous assembly (Additional file 4: Table S3). In examples we examined, in the VGP assemblies, we found single molecule PacBio reads that crossed the assembly or read depth gaps, or contig ends, found in the prior assemblies, without the presence of a real duplication of the gene(s) (Fig. 5), experimentally validating them as false duplications in the prior assemblies, and not computational errors.

Among non-coding sequences, long terminal repeats (LTRs) sequences of the zebra finch were reported to have expanded 2.5 times more than chicken [33, 43] and short interspersed nuclear elements (SINEs) were reported to be highly expanded in the platypus relative to other mammals [32]. However, we found 18,757 copies of LTRs (21% of the total) were false duplications in the previous zebra finch assembly and 140,279 copies of SINEs (6.1% of the total) were false duplications in the previous platypus assembly (Additional file 5: Table S4). In the previous Anna’s hummingbird assembly, 3 to 5% of LTRs, SINEs, and long interspersed nuclear elements (LINEs) were false duplications (Additional file 5: Table S4).

### Specific categories of genes have higher levels of false duplications

**Fig. 6:**
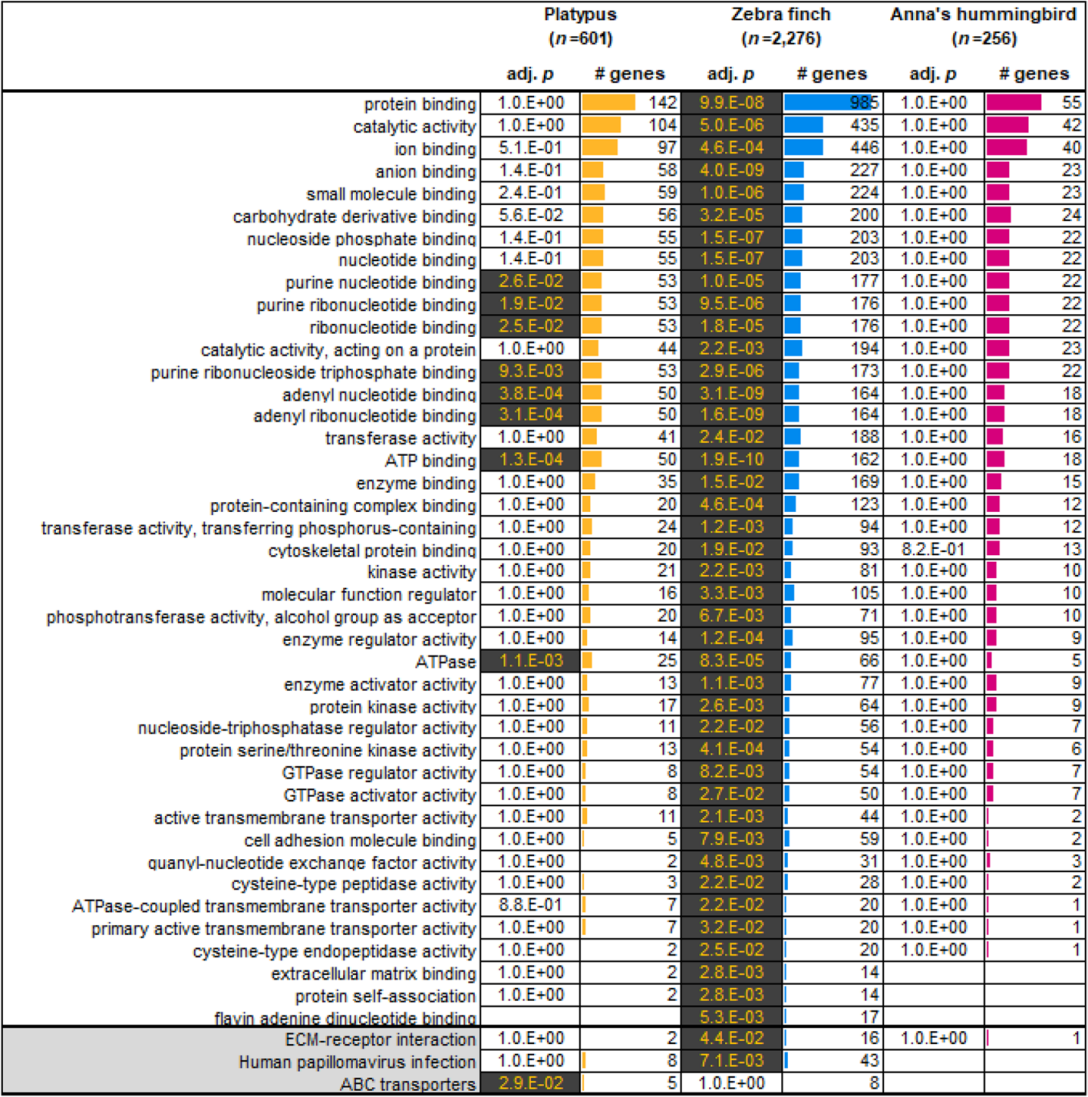
Gene ontology enrichment analysis of falsely duplicated genes. The gene ontology terms are shown in the first column. The number of falsely duplicated genes in the analysis is listed below the name of each species (*n*). The number of genes for each term (# genes) are represented in each species. The significant adjusted p-values (*P* < 0.05) are highlighted. The KEGG pathway terms are shown in the bottom

**Fig. 7:**
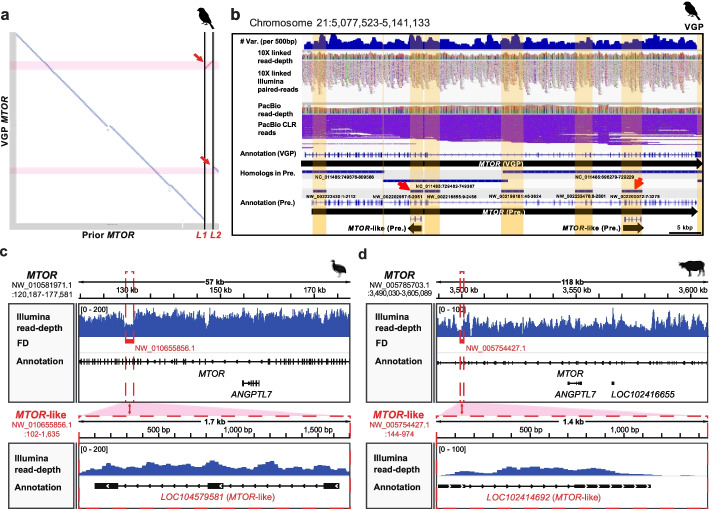
False duplication of the *MTOR* gene in vertebrate assemblies. **a** Alignment dot plot of the *MTOR* genes in the previous and VGP assemblies of the zebra finch. The alignment of two *MTOR*-like genes in the previous assembly is next to lines L1 and L2 and highlighted in pink. **b** Genome landscape of the *MTOR* gene in the VGP assembly. Heterozygosity density within 500-bp windows is shown at the top. The homologous regions of the previous assembly are represented with blue bars above each genomic position label. The falsely duplicated scaffolds including the *MTOR*-like gene in the previous assembly are shown with red arrows. **c** False gene gains of the *MTOR* gene in white-throated tinamou (GCF_000705375.1) and **d** water buffalo (GCF_000471725.1) assemblies. Scaffolds with false duplications (FD) of *MTOR*-like genes were aligned to parts of the original *MTOR* gene and indicated as red dot boxes in each panel

To determine if genes with false duplications belong to specific functional categories or are random in function, we performed GO enrichment analyses for the false gene lists of each species of the previous assemblies. We found 42 GO molecular function terms and 3 KEGG pathways were significantly enriched in the platypus and zebra finch falsely duplicated genes (Fig. 6). Out of these, there were 8 GO terms enriched in both species, and all 8 were nucleotide binding functions. Even though the Anna’s hummingbird results did not yield GO categories at our statistical cut off (*P* < 0.05), the highest ranking categories also included nucleotide binding functions (Fig. 6). The differences in significance values between species were correlated with the number of false duplications found, where more genes lead to greater significance. This included “ATP-binding” genes in both zebra finch and platypus, and 5 “ABC transporters” (ATP-binding cassette transporters) in platypus and 8 in zebra finch as false duplications (Additional file 6: Table S5). We observed the ATP-binding genes tend to show higher heterozygosity than the other genes (Additional file 2: Fig. S8). The ABC transporters are known as the one of the largest and oldest superfamilies, in diverse living organisms from prokaryotes to vertebrates, and play key roles in encoding membrane proteins that transport diverse metabolites [44, 45]. The extensive variation in this superfamily implies a high evolutionary divergence rate [46], leading to a higher prevalence in haplotype divergence [47]. In this family, we observed the *MTOR* gene, which regulates growth, metabolism, signaling, and disease with the kinase domain using ATP [48]; it was falsely duplicated in the previous assemblies of zebra finch, Anna’s hummingbird, and platypus (Fig. 7a, b; Additional file 2: Fig. S9). We found the heterozygosity of the regions falsely duplicated within the *MTOR* genes of all three species were higher (0.0160, 0.0050 and 0.0020 for zebra finch, hummingbird and platypus) than the regions that were not duplicated (0.0140, 0.0046 and 0.0014). Further, by applying purge_dups [31] (see details in the “Materials and methods” section), we found false gene gains of *MTOR* in other vertebrate species genome assemblies, including the white-throated tinamou and domestic water buffalo (Fig. 7c, d). These assemblies were generated with Illumina short reads only. Their *MTOR*-like harboring scaffolds and the homologous regions in original *MTOR* genes showed read coverages drops to the haploid-level.

### False duplication and annotation errors remaining in VGP assemblies

**Fig. 8:**
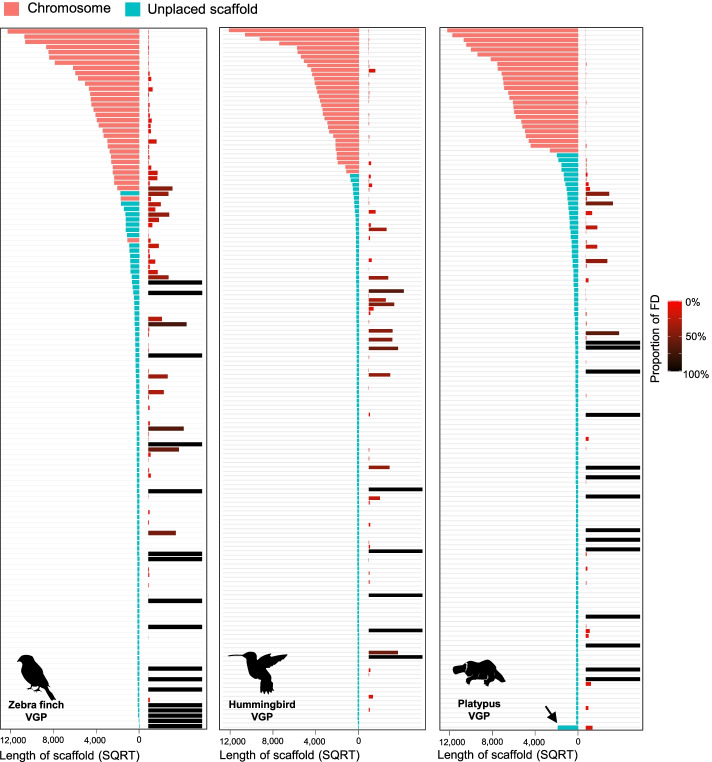
False duplications left in VGP assemblies. The left side of each graph shows the scaffold length of named chromosomes (pink) and unplaced scaffolds (turquoise). The right side shows the proportion of each scaffold that is falsely duplicated either within the same or different scaffolds, with color intensity indicating 0% (in red) to 100% (in black) falsely duplicated. Arrow: for the platypus, scaffolds < 40 kbp were concatenated into the one scaffold, where we found 20 scaffolds were completely duplicated among them

Although the amount of false duplications in the VGP assemblies was drastically lower than previous assemblies, here we found 74 to 119 scaffolds included false duplications, of which 5 to 34 (3–11% of the total number of scaffolds) were complete scaffold duplications (Fig. 8). From this error, we observed 1175, 119, and 94 genes of the zebra finch, hummingbird, and platypus were total or partial false duplications in the VGP v1.0 assemblies (Fig. 4g). False duplications were observed within both named chromosomes and unplaced scaffolds, with no discernable patterns in terms of chromosome (Additional file 2: Fig. S10a,c,e). However, for some small unplaced scaffolds (< 50 kbp), the proportion of their scaffolds as false duplications were large, with some cases where the entire scaffold was a false duplication (Additional file 2: Fig. S10b,d,f). This indicates that for the VGP assemblies, some unplaced scaffolds are simply the other haplotype or a homotype duplication the length of a raw read (1 to 50 kbp) with sequence errors.

**Fig. 9:**
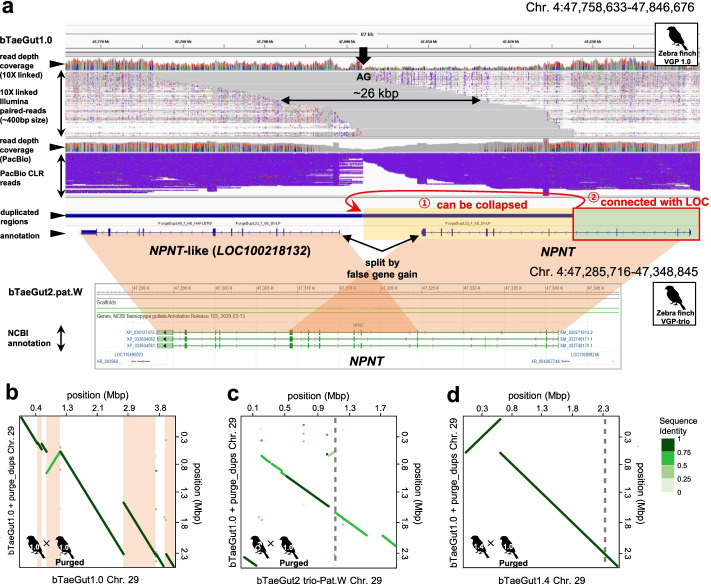
False duplications and their correction in the VGP zebra finch assembly. **a** The *NPNT* gene in the VGP zebra finch v1.0 assembly bTaeGut1.0 (first release) has the *NPNT*-like gene adjacent to it with an assembly gap (AG) and discordant 10X linked reads in this region. In contrast, the trio-based assembly (bTaeGut2.pat.W) had no *NPNT*-like gene, suggesting a false gene gain in bTaeGut1.0. The false duplication we found in this region was collapsed by purge_dups, and the falsely segmented gene structure was recovered. The VGP assembly v1.7 pipeline with purge_dups conducted before scaffolding prevented this false duplication (Additional file 2: Fig. S12). **b** Dot plot of alignment showing large ~1.9 Mbp false duplication of chromosome 29 (apricot) in the zebra finch VGP v1.0 pipeline assembly, bTaeGut1.0. **c** The large ~1.9 Mbp of duplications of chromosome 29 in bTaeGut1.0 were prevented in the trio-based assembly. **d** The 1.8 Mbp duplication was prevented with purging pre-scaffolding in bTaeGut1.4 using the VGP v1.7 pipeline. The boundaries of the scaffolds are represented as gray dashed lines

We manually verified examples and found some of the same type of errors seen in the previous assemblies, except the duplications were larger, presumably due to the longer read lengths and long optical maps of the VGP assemblies. An example was a false gene gain of *NPNT*, called *NPNT*-like (*LOC100218132*), on chromosome 4, named as such by the NCBI annotation pipeline applied to the VGP zebra finch 1.0 assembly (Fig. 9a). However, the false duplication structure caused 4 missing exons in the 5′ region of *NPNT* and 3 missing exons in the 3′ region of *NPNT*-like. Characteristic of the previous assembly, the false duplications were separated by an assembly gap, with discordant 10X linked reads and at haploid depth coverage. Other examples included those that contained non-coding sequence (Additional file 2: Fig. S11a) and those that contained false chimeric PacBio palindromic sequence the length or raw reads (7–17 kbp), both with 10X linked read depth gaps (Additional file 2: Fig. S11b,c). A case of a large duplication was on zebra finch chromosome 29, where 4 segments adding up to ~1.9 Mbp total were classified as false duplications using our criterion, making up ~45% of the assembled 4.2 Mbp microchromosome (Fig. 9b).

To verify whether many of these false duplications are due to false haplotype separation, we examined a VGP trio based assembly of another zebra finch individual [5]. This trio-based approach was recently developed with the goal of using parental short reads to separate out haplotype sequences of the child long reads, before contig assembly and scaffolding [5, 25]. We found that both the local *NPNT* (Fig. 9a) and the large ~1.9 Mbp of duplications of chromosome 29 were prevented in the trio-based assembly (Fig. 9c).

We sought a means to further prevent false duplications in non-trio based assemblies, as the individuals used in this study do not have available parental data. The VGP assemblies used in this study were produced with the VGP v1.0 pipeline [5], where heterotype duplications were removed by the purge_haplotigs algorithm [35] after scaffolding. In addition, many false duplications were detected and removed during manual curation [5]. As done for some later VGP assemblies of other species [5], but not directly tested on the same individual, we reassembled the zebra finch individual used for the v1.0 assembly here, but performed purge_dups before scaffolding contigs in the VGP v1.6 pipeline rather than afterwards in the VGP v1.0 pipeline. We also added a new tool called Merfin, to polish the assembly with long reads (https://github.com/arangrhie/merfin), a step that does not influence false duplications but improves base level accuracy. We called the update the VGP v1.7 pipeline. After reassembly, the *NPNT* and other false duplications were prevented (Additional file 2: Fig. S12, Additional file 7: Table S6). The 1.8 Mbp of false duplications on chromosome 29 were prevented, resulting in a smaller chromosome 29 consistent with removing false duplications manually (Fig. 9d). Overall, we observed a reduction from 1175 genes to 176 genes with false duplications (Fig. 4g), and reduction of 16 entire false duplicated scaffolds to 5 (Additional file 7: Table S6). These findings show that false duplications are still prevalent in some of the best assemblies, but have potential to be removed with improved haplotype phasing.

### Specific partitions of the genome with greater false duplications

Our above analyses focused on protein coding genes. Here we calculated the proportion of each genomic partition that was falsely duplicated. We found that in the previous and VGP assemblies, the intergenic regions had higher than expected false duplications based on the intergenic proportion of the genome, the introns lower than expected, and the exons no different than expected (Additional file 2: Figs. S13a and S14a,c). An exception was the VGP zebra finch assembly, where the introns and exons were higher than expected (Additional file 2: Figs. S13a). Among repetitive elements (LINEs, SINEs, LTRs, DNA, RNA, satellites,…), there were smaller differences relative to the expected proportions, with satellite repeats showing the highest above expected in some cases (Additional file 2: Figs. S13 and S14). These findings are consistent with the common knowledge that intergenic regions diverge at a greater rate than genic regions, thus having higher heterozygosity. We were surprised to find that introns have a lower proportion relative to exons, given their higher divergence as well.

### Assembly methods to minimize false duplication

We further investigated the false duplication rates generated by different assembly algorithms and steps that went into testing and developing the VGP assemblies, using the hummingbird data [5]. We calculated the proportion of *k-mer* duplications as a proxy for false duplications. As expected, the fully scaffolded, haplotype purged assembly had the least *k-mer* duplications (0.6% of the genome; Additional file 8: Table S7). For the specific steps, PacBio CLR assembly with FALCON-Unzip showed < 0.7% *k-mer* duplication (Additional file 8: Table S7). FALCON alone on PacBio CLR reads resulted in more *k-mer* duplications (1.4%). The Canu contig assembler on CLR generated the most *k-mer* duplications (5.4%). A hybrid Canu assembly of PacBio and Oxford Nanopore reads showed better performance (2.1%) than Canu alone. This suggests that although both FALCON and Canu are diploid-aware assemblers, the haplotype resolving algorithm in FALCON-Unzip has a greater advantage in preventing false duplications. The Illumina short read assemblies generated with the 10X Genomics linked reads Supernova2.2 assembler and paired end reads with SOAPdenovo produced high *k-mer* duplications of 10.1% and 5.2%, respectively, even though both algorithms attempt to phase haplotypes [18, 49]. In general, the scaffolding steps in the VGP pipeline with 10X linked reads, optical mapping, and Hi-C reads did not suppress false duplications, whereas purge_haplotigs and purge_dups were more effective to eliminate false duplications before scaffolding. False duplications of Bionano maps could also be introduced. But, most false duplications occur in the contigging step, which are then they are propagated in the scaffolding steps. Therefore, supporting our prior conclusions [5], there is a need for the haplotype resolving early in the assembly process, in contigging step.

We also further investigated the presence of false duplications to the other recent assemblies produced originally outside of the VGP group (Additional file 2: Fig. S15). We analyzed the emu, which included one assembly generated with Illumina short reads (ASM1339679v1) [50] and another generated with PacBio CLR reads, and scaffolded with 10X and Hi-C reads (ZJU1.0) [51]. Surprisingly, we found more false duplications in recent long-read assembly made by FALCON-Unzip and purge_haplotigs (14Mbp; 1.1% of the assembly) than the short-read one (1Mbp; 0.1% of the assembly) made by AllPaths-LG [52]. *K-mer* profiles of these assemblies show that the heterozygosity is not significantly different between the individuals (Additional file 2: Fig. S16), and thus this cannot be the explanation for the differences in the assemblies. We know that purge_haplotigs only removes false duplications that are on different contigs, whereas purge_dups also removes false duplications within contigs/scaffolds. We are working with the developers of the emu assembly to clean up these false duplications potentially with purge_dups. Overall these findings show that a combination of assembly methods and level of heterozygosity are key factors contributing to and preventing false duplications.

## Discussion

In this study, we found that the primary characteristics of false duplications include (1) half depth of read coverage for heterotype duplications, or very low depth for homotype duplications; (2) presence of gaps between duplicated pairs on the same scaffold; (3) discordant or spanned linked read pairs used for scaffolding, whenever 10X or other types of paired reads of a DNA fragment were used; and (4) 1 copy *k-mers* for heterotype duplications. Some of these characteristics have been reported in other studies prior to the VGP effort [3, 5, 22, 35], but not in a systematic manner of comparing previous and new assemblies that attempted to remove false duplications as reported here. The false duplications were highest in the previous Sanger-based assemblies and lowest in the VGP PacBio-based long-read assemblies that purged them before scaffolding or in a VGP trio PacBio-based long-read assembly that sorted haplotype reads before contig and scaffold generation. One major source of the false duplications was a near doubling in the level of heterozygosity in the false duplicated regions compared to the rest of the genome. Further, the species with the highest heterozygosity, the zebra finch, had the highest proportion of false duplications in the previous and VGP assemblies. Another major source was sequencing error, in both previous and VGP assemblies.

These false duplications led to mis-annotations as false gene, exon, and chimeric gene gains. When the duplication is created, the inserted allelic sequence results in annotation of two similar genes or one original and several fragmented genes. These types of false gains were made in genes involved in important phenotypes, leading to misinterpretations in downstream analysis [21]. For example, false gene gains reduce one-to-one orthologs, which are used in comparative genomics and phylogeny. When false gains occur in an expanded gene family of closely related genes, this leads to false-positive cases of gene family expansions and gene duplications as we report here, others previously [53], and in a companion study on the oxytocin family of receptors [54]. For phylogeny, these duplications create false orthologs or indels in genes that weaken gene- and species-inferred relationships. This can be made worse with multiple false duplications of genes with closely related paralogs, such as the overestimated LTR expansion in the zebra finch [33, 43], and ATP-binding gene family across species. Our findings indicate that caution should be taken when interpreting gene family expansion in assemblies generated without haplotype phasing and checking for false duplications.

Our findings that heterotype false duplications are much higher than homotype indicate that proper haplotype separation is still a current problem in genome assembly, even when they have been greatly reduced in the VGP assemblies. The VGP 1.6 trio pipeline removes more heterotype false duplications [5], but it requires parental sequence data to sort haplotypes, and parents will not be available for all individuals. Scanning regions around gaps with reads and *k-mer* profiling, and discordantly mapped Illumina linked short reads or disconnected PacBio long reads should be helpful in identifying false duplications in any assembly. However, the best way to prevent these we propose would be to improve haplotype phasing of raw reads without parental data, remove reads with sequence errors before assembly, and generate complete diploid genome assemblies.

The VGP group is constantly updating its sequencing and assembly pipeline to create a genuine blueprint for assembly of complex and large genomes as found among vertebrates. Doing so requires in-depth evaluation of assemblies, as done in this study. In the VGP assembly pipeline, the CLR data type of PacBio sequencing was recently replaced in 2021–2022 with the closed circular sequence (CCS) high fidelity (HiFi) read data type [5, 55], which reduces the base-pair error rate without the need for short-read Illumina polishing. We expect these new HiFi reads to also reduce the false duplications due to sequence errors, and it may allow better separation of haplotypes; promising alternatives include recent assemblers that use Hi-C data to phase haplotypes before or during contig assembly, FALCON Phase [56], and hifiasm (Hi-C) reported in a preprint [57]. The HiFi sequence read lengths, however, are currently ~20% shorter (15–20 kbp) than CLR and thus may lead to less contiguity across real duplications longer than the read lengths. Our findings emphasize that creating haplotype-phased reference genome assemblies free of false duplications should be a fundamental requirement of future genomics and biology.

## Conclusions

We found widespread false duplications in genome assemblies and they are the source of various misinterpretations of gene gains and gene family expansions. The main source of the false duplications were algorithms that did not properly handle increased heterozygosity or sequencing errors. In the several cases, long-read based assemblies tend to have lower *k-mer* duplication than short-read ones, and haplotype resolving algorithm played a key role in preventing false duplicated contigs. This study emphasizes that a systematic effort in both sequencing and assembly steps is crucial to suppress false duplications, and the need for cautious analyses on gene gains.

## Materials and methods

### Assemblies and read data

The primary assembly of the previous and VGP version of the male zebra finch, female Anna’s hummingbird, and female and male platypus were downloaded from NCBI by ftp along with their assembly statistics, gaps, repeats, and annotation data (Additional file 1: Table S1). For the VGP assemblies, we included both the primary and alternate pseudo-haplotype sequences. The raw reads used for the previous assemblies of the zebra finch and platypus generated by Sanger sequencing were not available to download from the Sequencing Read Archive (SRA) on NCBI. However, the raw Sanger reads of the previous version of the platypus assembly was in the Trace Archive in NCBI (https://ftp.ncbi.nlm.nih.gov/pub/TraceDB/ornithorhynchus_anatinus/). We downloaded all “.anc” and “.fasta” files and extracted the reads that were submitted by “WUGSC” for platypus assembly. These Sanger reads from the older assembly and the 10X linked reads of the new assembly were used to quantify whether the duplications were due to individual differences between previous and VGP assemblies or real false duplications. The PacBio CLR and 10X raw reads used to generate the VGP assemblies were downloaded from the VGP Genome Ark (https://vgp.github.io/genomeark/).

### Identifying false duplications

#### Candidate duplications from sequence similarity

We identified false duplication candidates by sequence similarity in whole genome alignments between the previous and VGP assemblies and self-alignment of an assembly to itself. We used Cactus [37, 58] to generate whole genome alignment across assemblies with the default options and HAL [59] to transform the Cactus results into a readable multiple alignment format with “--maxBlockLen 1,000,000 --noAncestors --refGenome” (VGP assembly as reference) parameters. One to many homologs between two assemblies of the same species were then considered as potential false duplication candidates. Since the Cactus alignment contained very short sequences (<20 bp) in alignment blocks, we filtered out blocks shorter than 20 bp or query sequence coverage of less than 80% to avoid spurious alignments. Self-alignment was performed with Minimap2 [36] with the “-xasm5 –DP” option on for assembly alignment mode, after segmenting contigs by “N”-base gaps. Purge_dups was then used to find false duplications [31] with “-2” option following the guideline of purge_dups (https://github.com/dfguan/purge_dups). We used a purge_dups version that we asked the developers to modify (“add_loc” branch in github of purge_dups; https://github.com/dfguan/purge_dups/tree/add_loc) to output the pair-wise homologous loci for each false duplication found.

#### Filtering true duplications

False duplication candidates were distinguished from true haplotype specific duplications using 10X linked read alignments; it was difficult to map PacBio CLR reads to the previous assemblies, as the length of the majority of the contigs of the prior assemblies (e.g., 1~3 kbp) was shorter than the PacBio read lengths of the VGP assemblies (e.g., ~10–17 kbp). The paired-end reads from the linked reads were aligned with EMA v0.6.2 [60] using the barcodes default option, and BWA v0.7.17 [61] without the barcodes with parameters “-p -M -R ‘@RG\tID:rg1\tSM:sample1’” options following guideline of EMA. Duplicate reads were marked by Sambamba v0.7.1. Coverage distribution across the entire assembly was extracted using samtools [62]. False duplication candidates from purge_dups self-alignments were further processed using the remainder of the purge_dups pipeline, which included generating coverage distributions.

Candidates from the Cactus alignments were similarly filtered using the same read depth threshold as in purge_dups. Any duplications with lower than half the diploid read depth of coverage were further considered. We then applied two additional criteria: (1) presence of a scaffolded gap or read depth-gap between a duplicated pair and (2) discordant read pair alignments. A depth-gap is defined as a region with no read alignments between duplicated pairs, which occurs from incorrect gap-filling or incorporation of reads with sequencing errors during assembly (Additional file 2: Fig. S3). A discordant read pair was defined when the insert size between the pairs is unexpectedly large (>550 bp; mean insert size of 10X read in this study) or mapped to another scaffold as in Kelley and Salzberg [3]. We required both the presence of discordant reads and concordant reads to align, where one end from a discordant read pair and concordant read pair aligns to the identical flanking region (~550 bp) of a duplication, while the other end aligns to each of the homologous duplications.

#### Classifying heterotype and homotype duplications

The filtered false duplications were further classified based on *k-mer* analysis (considering genome size, *k* = 20 for all three species). We extracted 20-mers from the assemblies and 10X linked reads using Meryl [24] and performed Merqury [24] analysis to obtain the *k-mer* spectrums, using the non-trio mode for pseudo-haplotype assembly. Using the *k-mer* spectrum, we defined erroneous *k-mers* as those found in the assembly with read multiplicity lower than 6x, 3x, and 18x in the previous assemblies of zebra finch, hummingbird, and the platypus, and 3x, 3x and 10x for the VGP assemblies, respectively. These are low-multiplicity *k-mers* in the *k-mer* spectrum, made by sequencing errors [24, 25]. Likewise, any non-erroneous *k-mer* found once in the assembly was defined as a single-copy *k-mer*. We classified false duplications as heterotype when both of the duplicated pairs had single-copy *k-mers* with average read depth higher than sequencing error read depths, which was 5x, 8x, and 22x for the previous assemblies and 2x, 2x, and 9x for the VGP assemblies of the zebra finch, hummingbird, and the platypus, respectively (same principle with erroneous *k-mers* identification), otherwise as homotype duplication, which had no single-copy *k-mer* found on either side of the duplication or one duplication of the pair had read depth below heterotype duplication levels.

### Evaluating false duplications

PacBio CLR reads were mapped to both the previous and VGP assemblies using Minimap2 [36] with the preset “-ax map-pb”. Sanger reads of platypus were also mapped to the previous assembly using Minimap2 with “-ax map-pb” and used for further read coverage evaluation. Since the coverage distribution of Sanger reads showed a unimodal distribution at 1x coverage, we defined the threshold of haploid-level coverage for Sanger reads as the mean depth-coverage of the total assembly*0.75. The mapped reads on each assembly were visualized with IGV [63]. Duplications found in the VGP assemblies were aligned to their counterpart assembly and visualized with D-Genies [64]. The location of false duplications in the VGP assemblies was visualized by karyoploteR [65].

The heterozygosity of assemblies, including each corrected FD and correctly assembled region, was calculated as the number of variants divided by the length of the region, with 1000 bootstrapping replicates to generate a distribution for a Student’s *t*-test between those regions. To calculate heterozygosity in the region of the introduced FD in the VGP assemblies, we masked false duplications as “N”s, then the variant was estimated from newly mapped 10X linked reads onto the masked assembly, followed by the same bootstrapping and statistical approach as used above. Samtools and bcftools were used for variant calling with the multiallelic model. We filtered-out variants with biased alleles, i.e., we only considered the locus if the proportion of major and minor alleles were in >25% and <75%. The sequence error rate of each duplicated and correct region was calculated by dividing the number of erroneous *k-mers* by the total number of *k-mers* found. The distributions of sequencing error rate for duplicated and correct regions were also generated by 1000 bootstrapping replicates, and a Student’s *t*-test was performed on those distributions.

### Identification of false gene gain annotation errors

We calculated the number of protein coding genes affected by false duplications, defined as regions with duplicated sequences that overlapped with the CDS regions of an assembly. The Refseq annotation of NCBI was used and only the longest CDS of all isoforms generated from each gene was used. The genes influenced by false duplications were classified into three types: (1) false gene gain (FGG) in which a gene was falsely duplicated almost entirely or partially over 50% of the CDS length; (2) false exon gain (FEG) of one or more exons within the same gene; and (3) false chimeric gain (FCG) in which duplicated exons from one gene were inserted into another gene. FGG, FEG, and FCG were included only when at least one coding exon of a gene completely overlapped the false duplication. To visualize the example cases of mis-annotation, GSDS 2.0 [66] was used. Intergenic regions were defined as the remaining regions excluding CDS and intron.

To search for possible false duplications in non-coding repetitive elements, we counted the number of LTRs, SINEs, and LINEs affected by false duplications using NCBI repeat information generated by repeatMasker [67]. Then, the relative proportion of false duplication on each genomic partition was calculated by the difference between observed and expected proportion. The observed is the proportion of each genomic partition containing false duplications (Σ feature length overlapped with false duplication/total false duplication length) of each assembly. The expected is the normal proportion of each genome partition (Σ feature length/total assembly length). The differences between observed and expected genomic partitions were tested by one-way analysis of variance (ANOVA).

In the platypus, we also searched false gene gains of the V1R family in the same manner as above. We checked for 267 V1R genes for potential false gene gains in the previous assembly of the platypus, which included “ORNANAV1R” in the gene symbol.

### False duplication correction using the VGP pipeline v1.7

We reassembled the zebra finch assembly using a variation of the VGP v1.0-1.6 pipelines, which we called the VGP v1.7 pipeline. Aside from software updates, the two main differences with respect to the VGP v1.0 pipeline [5] are (1) purge_haplotigs was replaced by purge_dups, for more effective purging of false haplotype and homotype duplications; (2) purging was done after contiging, as opposed to after scaffolding and polishing; and (3) during the final Arrow polishing step, variant calls were filtered with Merfin (https://github.com/arangrhie/merfin), to avoid introducing erroneous *k*-mers in the assembly. This resulted in the following assembly steps: (1) FALCON-Unzip contig assembly; (2) purge_dups to purge false duplications in the primary assembly, and place them in the alternate assembly; (3) scaffolding the primary assembly with 10X linked reads and scaff10X software; (4) scaffolding with Bionano optical maps and Bionano solve software; (5) scaffolding with Arima Genomics Hi-C and Salsa v2.2 software; (6) polishing with long reads using Arrow and filtering the variant calls with Merfin; and (7) a final polishing with longranger aligner and freebayes. We added the assembled mitochondrial genome prior to the polishing steps to prevent overpolishing of NUMTS in the nuclear genome. We compared this VGP 1.7 assembly (bTaeGut1.4) with the zebra finch VGP v1.0 pipeline (bTaeGut1.0; GCF_003957565.1) by alignment using Cactus [37]. Based on the regions of false duplication, we found in bTaeGut1.0, the homologous regions of false duplication were extracted by Hal [59]. We calculated the uncorrected amount of false duplications in bTaeGut1.4 from each false duplication in bTaeGut1.0 as follows: Given a length of homologous sequence *H* of a false duplication (*FD*) from new (*v1.7*) and prior (*v1.0*) VGP zebra finch in an alignment block, an uncorrected false duplication was calculated as *uncorrected FD* = Σ*H*_*v1.7*_ − (Σ*H*_*v1.0*_ − *FD*). If the uncorrected false duplications were ≤ 0 bp, we regarded that false duplication was corrected in the bTaeGut1.4 assembly.

### Duplicated k-mers in different hummingbird assembly approaches

We calculated *k-mer* duplications for each experimental hummingbird assembly generated by Rhie et al. [5] for assessing the relative magnitude of introducing or removing false duplications by various assembly algorithms and steps. The assemblies are available in GenomeArk prefix on “s3://genomeark/working/release1/scaffolding/” named as “bCalAnn1_c1.fasta.gz,” “pac_fcn_p.fasta.gz,” “pac_nano_canu.fasta.gz,” “pac_canu.fasta.gz,” “10x_spnv2_hap1.fasta.gz,” “ill_soap.fasta.gz,” and the primary VGP assembly of bCalAnn1.0. 10X linked reads of the hummingbird were used for calculating *k-mer* multiplicity. Meryl [24] and Merqury [24] were performed to obtain intermediate data points for analyzing *k-mer* duplications, with default options. *K-mer* duplications were counted by “false_duplications.sh” in the Merqury package.

### Gene ontology enrichment test for falsely duplicated genes

We tested gene ontology enrichment for the false gene gains, false exon gains, and false chimeric gene gains of each prior assembly. We used g:Profiler [68] on the web (https://biit.cs.ut.ee/gprofiler/gost) for functional profiling of these genes. Because the many false gene gains were fragmentary artifacts such as “-like” gene, we converted the gene symbol of these false gene gains using the original gene product name. g:Profiler supported the zebra finch and platypus in organism parameter selection, but the hummingbird was not supported. We thus selected the organism parameter “zebra finch” for the hummingbird by considering the closest phylogenetic distance of species listed in the database. Significance was calculated by g:SCS with a threshold of *P* < 0.05. The list of ATP-binding genes were made up by referring to vertebrate ATP-binding genes in AmiGO 2 (http://amigo.geneontology.org/amigo/). The control gene set was constructed by randomly choosing genes as the same number of ATP-binding genes for each species. Heterozygosity of the genes were calculated in each VGP assembly using the same method above. A significant difference of heterozygosity was tested by one-sided Wilcoxon rank-sum test. The *MTOR* gene in the VGP assemblies of the three species were broken down to the homologous regions of the false duplications and the correct single copy regions of previous assemblies, for calculating heterozygosity difference.

### Falsely duplicated MTOR genes in other reference genome assemblies

To test for possible false duplications of the *MTOR* gene in other published genome assemblies of vertebrates, we extracted 449 RefSeq annotated genomes of 330 vertebrate species from NCBI and found 38 assemblies have the original *MTOR* gene and at least 1 *MTOR*-like genes, respectively. We parsed the genic sequences of each gene from each assembly, aligned them for each species by using LAST [69], checked the *MTOR*-like harboring scaffolds were fully aligned to parts of the genic region of the *MTOR* genes, calculated proportions (>50%) of lengths of *MTOR*-like genes per scaffold with the duplicated genes, and considered the qualities and quantities of sequencing reads used to the generate assemblies. Following the above steps, we identified 4 assemblies of 4 species (*Bubalus bubalis*, *Tinamus guttatus*, *Scleropages formosus*, and *Bufo gargarizans*) that have scaffolds with duplicated *MTOR*-like genes. For the 4 candidate assemblies, we mapped raw sequencing reads used to generate each assembly and applied purge_dups and assessed whether they are false duplications. We discovered 2 assemblies out of the 4 assemblies (i.e., species; white-throated tinamou [*Tinamus guttatus*] and domestic water buffalo [*Bubalus bubalis*]), contained erroneous scaffolds with falsely duplicated *MTOR*-like genes (Fig. 7c, d).

### False duplications in emu assemblies

The assembly and raw sequenced data of emu were collected from NCBI Assembly for both previous (GCA_013396795.1) and recent (GCA_016128335.1) assemblies. The short reads generated from both individuals of the assemblies were available in NCBI SRA. We mapped the Illumina reads constructed by the 800bp paired end library (run number: SRR9946765, SRR9946766, SRR9946768, SRR9947049, SRR9994342, SRR9994343, SRR9994348, SRR9994349, SRR9994351) to the previous assembly using Minimap2 with preset “-ax sr.” We mapped the 10X linked reads of the other assembly (run number: SRR11971566) to the recent assembly with the same step for 10X linked read mapping above. We ran the same pipeline for identifying false duplications in both assemblies, with filtering true duplication as above.

## Supplementary Information

Additional File 1: Table S1. Statistics of previous and VGP assemblies.

Additional File 2: Figure S1-S16.

Additional File 3: Table S2. Mis-annotations caused by false duplications in both previous and VGP assemblies.

Additional File 4: Table S3. False duplication of V1R family genes in the previous platypus assembly.

Additional File 5: Table S4. False duplications on transposable elements in previous assemblies.

Additional File 6: Table S5. Gene ontology enrichment analysis for the false gene gains, false chimeric gains and false exon gains in previous assemblies.

Additional File 7: Table S6. Reduction of false duplications in the reassembled bTaeGut1.4 zebra finch genome with the VGP v1.7 pipeline.

Additional File 8: Table S7. Proportion of k-mer duplication measured for each assembly strategy.

Additional File 9. Review history.

## Data Availability

Genome data of zebra finch, Anna’s hummingbird, and platypus’ NCBI accession numbers are available in Additional file 1: Table S1. 10X linked read and PacBio CLR read data for VGP assemblies [5] are publicly available at GenomeArk (https://vgp.github.io/genomeark/) for each species [70]. The trio version of the zebra finch assembly is available in NCBI (GCF_008822105.2) [70]. Raw sequence reads of the prior platypus assembly are available in the NCBI trace archives [71]. The assemblies used for the *MTOR* gene duplication analyses are available in NCBI (tinamou: GCF_000705375.1 [72], domestic water buffalo: GCF_000471725.1 [73], Asian arowana: GCF_001624265.1 [74], Asiatic toad: GCF_014858855.1 [75]) Raw sequence reads of the tinamou, domestic water buffalo, Asian arowana, and Asiatic toad are available in NCBI SRA (SRR952232-SRR952240 [72]; SRR5562991-SRR5562996 [73]; SRR952232-SRR952240 [74]; SRR11624379, SRR11624380 [75]). Emu assemblies are available in NCBI (prior: GCA_013396795.1 [76], recent: GCA_016128335.1 [77]). Illumina and 10X linked sequence reads of the Emu assemblies used in this study are available in NCBI SRA (Illumina: SRR9946765, SRR9946766, SRR9946768, SRR9947049, SRR9994342, SRR9994343, SRR9994348, SRR9994349, SRR9994351 [76], 10X linked: SRR11971566 [77]). All source code used for identifying false duplications is available in both Github platform, https://github.com/KoByungJune/FalseDuplication [78] and Zenodo, 10.5281/zenodo.6510546 [79]. The analysis methods applied in this study for identifying false duplications are freely available in the above source code.
