# Supplementary material for: Widespread false gene gains caused by duplication errors in genome assemblies
Source: Genome Biol. 2022 Sep 27;23:205. doi: 10.1186/s13059-022-02764-1 (PMC9516828; doi:10.1186/s13059-022-02764-1)
Supplement: Supplementary file 2 — Additional File 2: Figure S1-S16. [file 13059_2022_2764_MOESM2_ESM.docx]

**Supplementary Information**

**Widespread false gene gains caused by duplication errors in genome assemblies**

**Additional File 2: Figure S1-S16**

Byung June Ko^1,+^, Chul Lee^2,+^, Juwan Kim^2^, Arang Rhie^3^, DongAhn Yoo^2^, Kerstin Howe^4^, Jonathan Wood^4^, Seoae Cho^5^, Samara Brown^6,7^, Giulio Formenti^6^, Erich D. Jarvis^6,7*^ and Heebal Kim^1,2,5*^

^1^Department of Agricultural Biotechnology and Research Institute of Agriculture and Life Sciences, Seoul National University, Seoul, Republic of Korea

^2^Interdisciplinary Program in Bioinformatics, Seoul National University, Seoul, Republic of Korea

^3^Genome Informatics Section, Computational and Statistical Genomics Branch, National Human Genome Research Institute, National Institutes of Health, Bethesda, USA

^4^Wellcome Sanger Institute, Cambridge, UK

^5^eGnome, Inc, Seoul, Republic of Korea

^6^Laboratory of the Neurogenetics of Language, The Rockefeller University, New York, NY, USA

^7^Howard Hughes Medical Institute, Chevy Chase, MD, USA

+These authors contributed equally: Byung June Ko, Chul Lee

*Corresponding authors

Corresponding author e-mail: Erich. D. Jarvis, ejarvis@mail.rockefeller.edu; Heebal Kim, heebal@snu.ac.kr

# Supplementary Figures

**
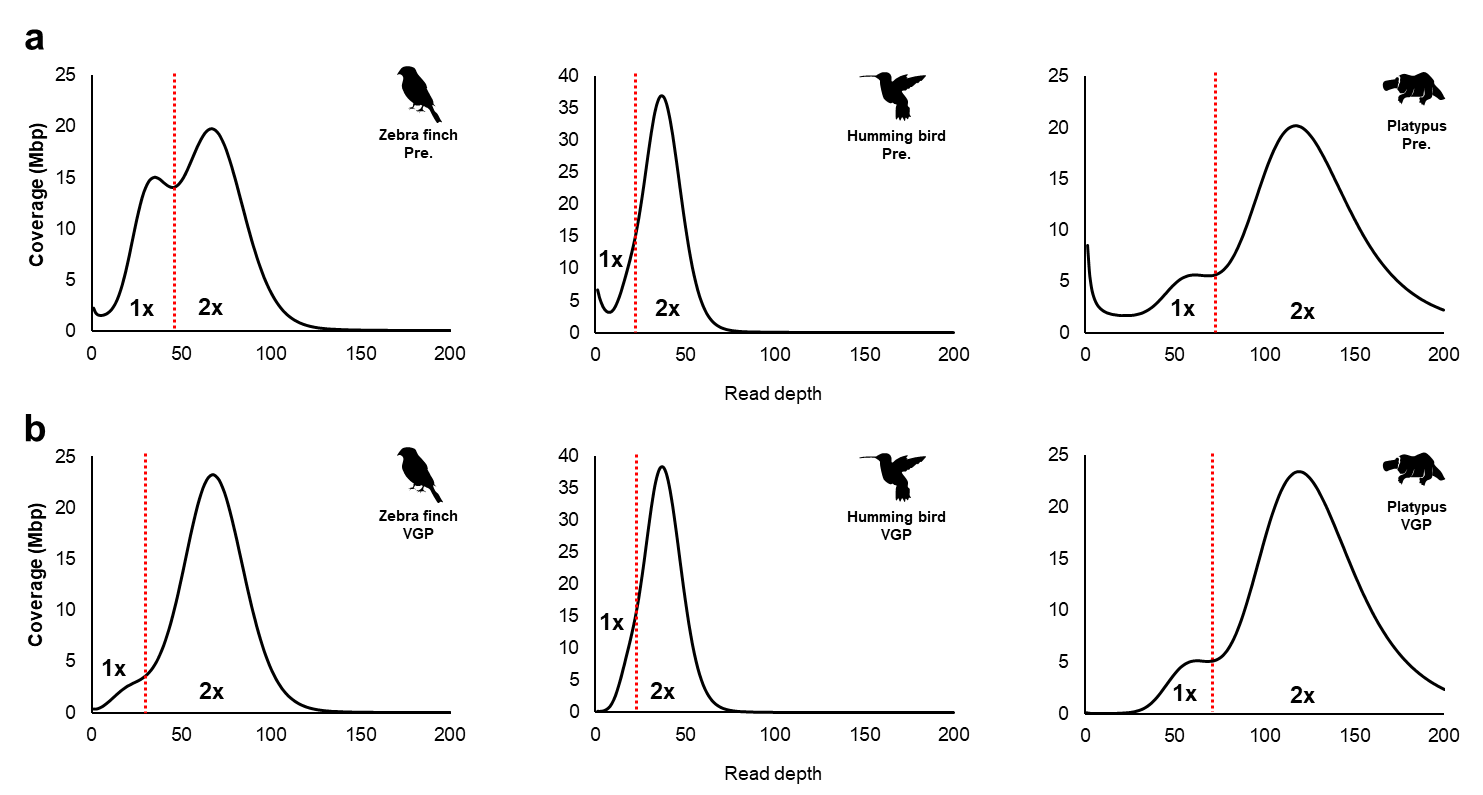
**

**Fig. S1 Depth-coverage profiling of all assemblies. a,** Prior assemblies. **b,** VGP assemblies. The 10X linked read depth-coverages of every site is summarized as a distribution. The red line shows the threshold of depth-coverage that we used to determine false duplications (to the left of the red line). The bimodal distribution in the zebra finch and the platypus assemblies are caused by highly heterozygous regions.


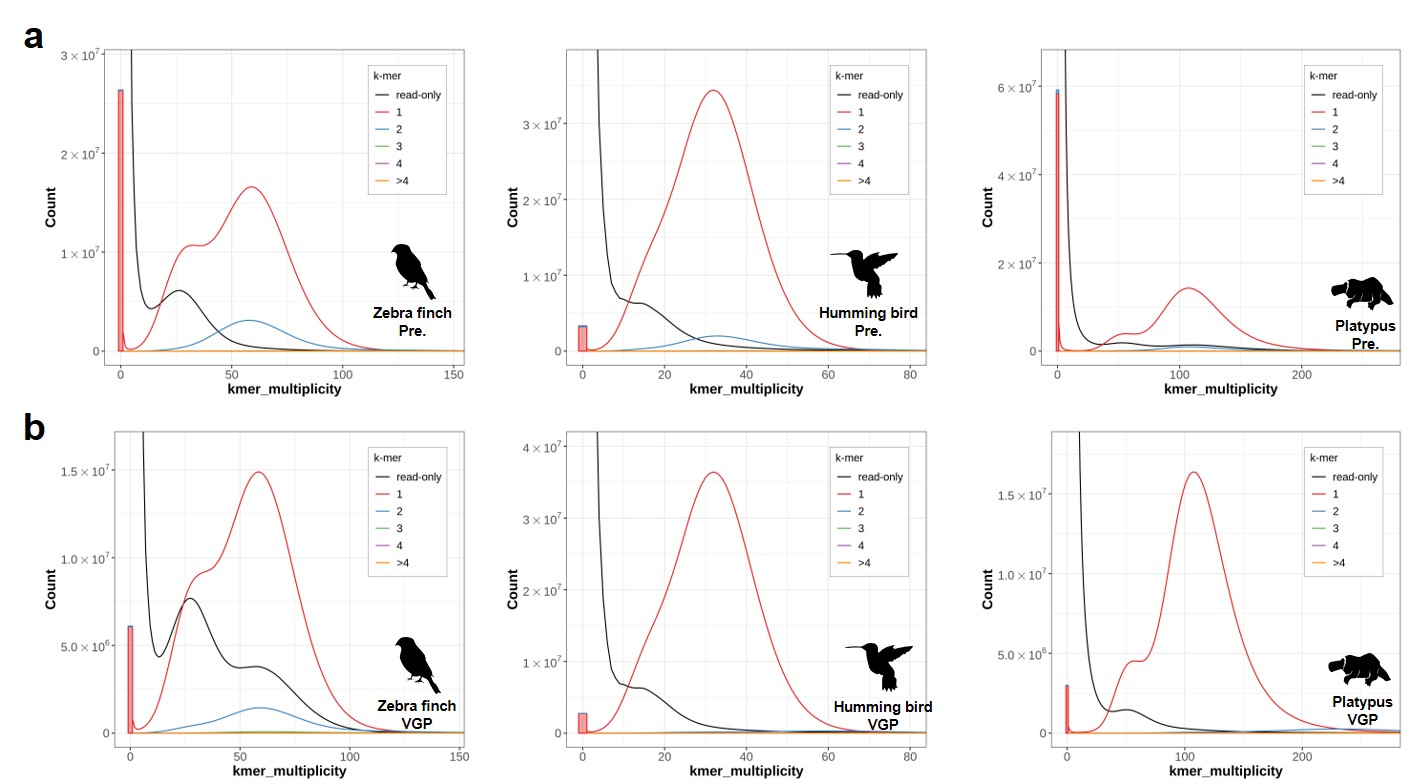


**Fig. S2 *K-mer* profiling for all assemblies. a,** Prior assemblies. **b,** VGP assemblies. From the sequences of 10X linked reads and assemblies, *k-mer* multiplicity was calculated. The x-axis is the *k-mer* multiplicity calculated from the raw reads, the y-axis are the counts, and the numbers in the boxes represent the *k-mer* multiplicity found in the primary pseudo-haplotype assembly. *K-mer* multiplicity of 2 copies or higher under the area of single copies (red) are overly represented as false duplications.


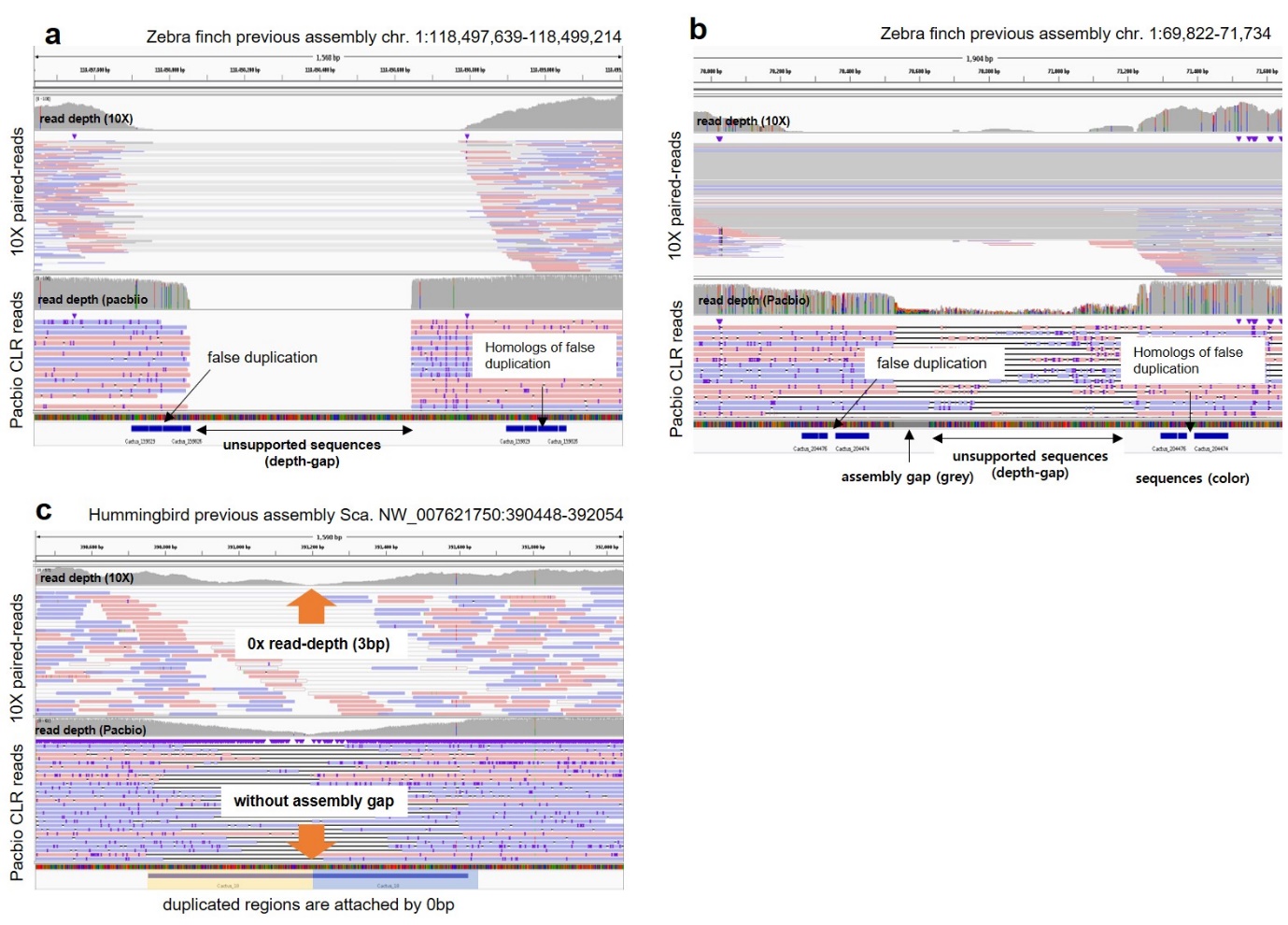


**Fig. S3 Unsupported sequences with or without assembly gaps.** 10X linked reads are shown as paired read alignments, and the PacBio CLR read alignments below them, along with the depth coverage of the respective read data. **a,** Unsupported sequence with a depth-gap but no assembly gap, between a false duplication. **b,** Unsupported sequence observed with an assembly gap, between a false duplication. **c,** Unsupported sequences with 0 bp between a false duplication. Unsupported sequences in the assembly were identified with 10X linked reads with no depth of coverage.


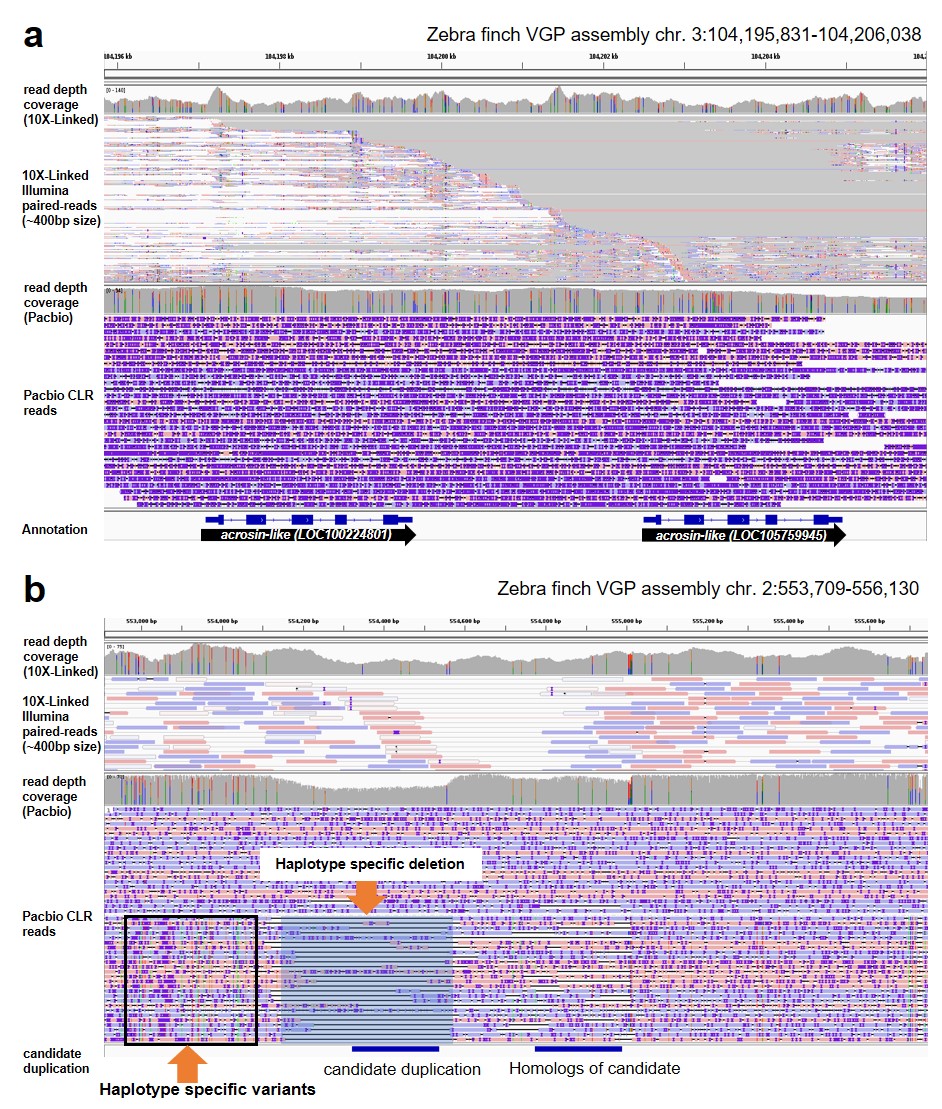


**Fig. S4 True duplication in a VGP assembly.** 10X linked reads are shown as paired read alignments above the PacBio CLR read alignments, along with the depth coverage of the respective read data. **a,** True gene duplication of the acrosin (*ACR*) gene in the zebra finch. The 10X linked read alignment shows discordant read alignments but has no signature of a depth-gap or decreased read-depth coverage. PacBio CLR reads alignments connect these duplicated genes with no gaps, as single molecule reads, and no unsupported sequence. **b,** True haplotype specific sequence duplication with lower read depth in the zebra finch**.** A candidate duplication was identified as a true genomic duplication, but in one haplotype. The 10X linked read alignment shows discordant read alignments but has no signature of a depth-gap. Half of the PacBio CLR read alignments show the allele specific duplication, while the other half show a deletion on one of the two alleles.

**
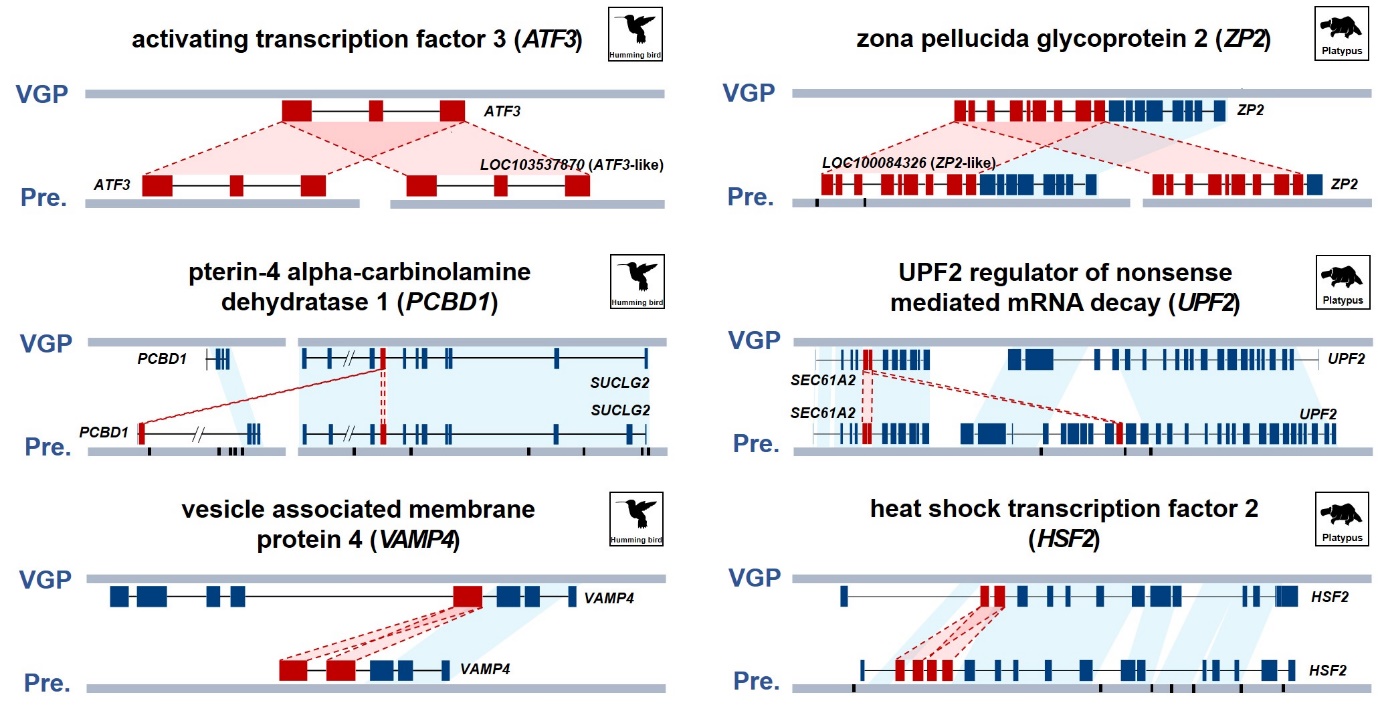
**

**Fig. S5 Cases of false gene gain annotations in the prior hummingbird and platypus assemblies.** Top row of each alignment shows the VGP 1.0 assembly structure and annotation. Bottom row shows the previous assembly structure and annotation. The red lines represent boundaries of the false duplicated exons in the prior assemblies that are correctly assembled in the VGP assembly. The blue boxes represent the correctly assembled exons in both the previous and VGP assemblies. The black bars represent the assembly gaps in the scaffolds.


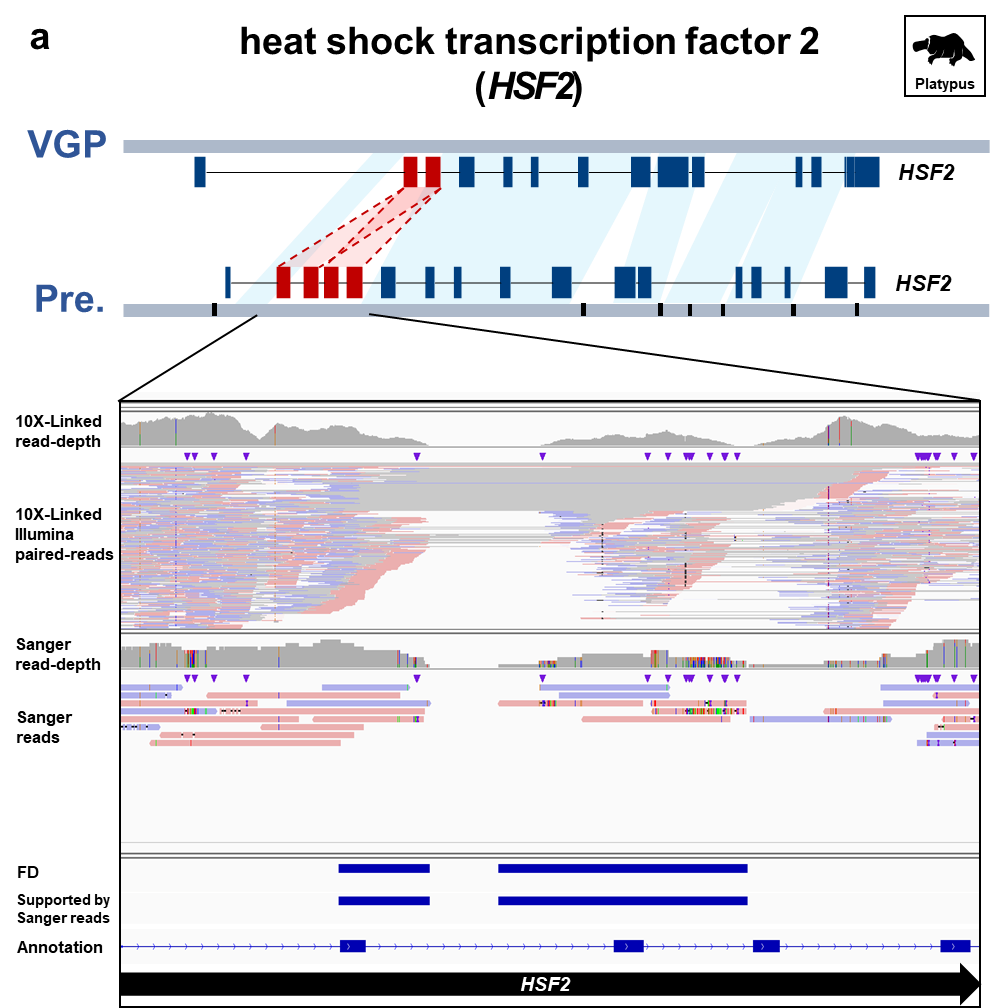

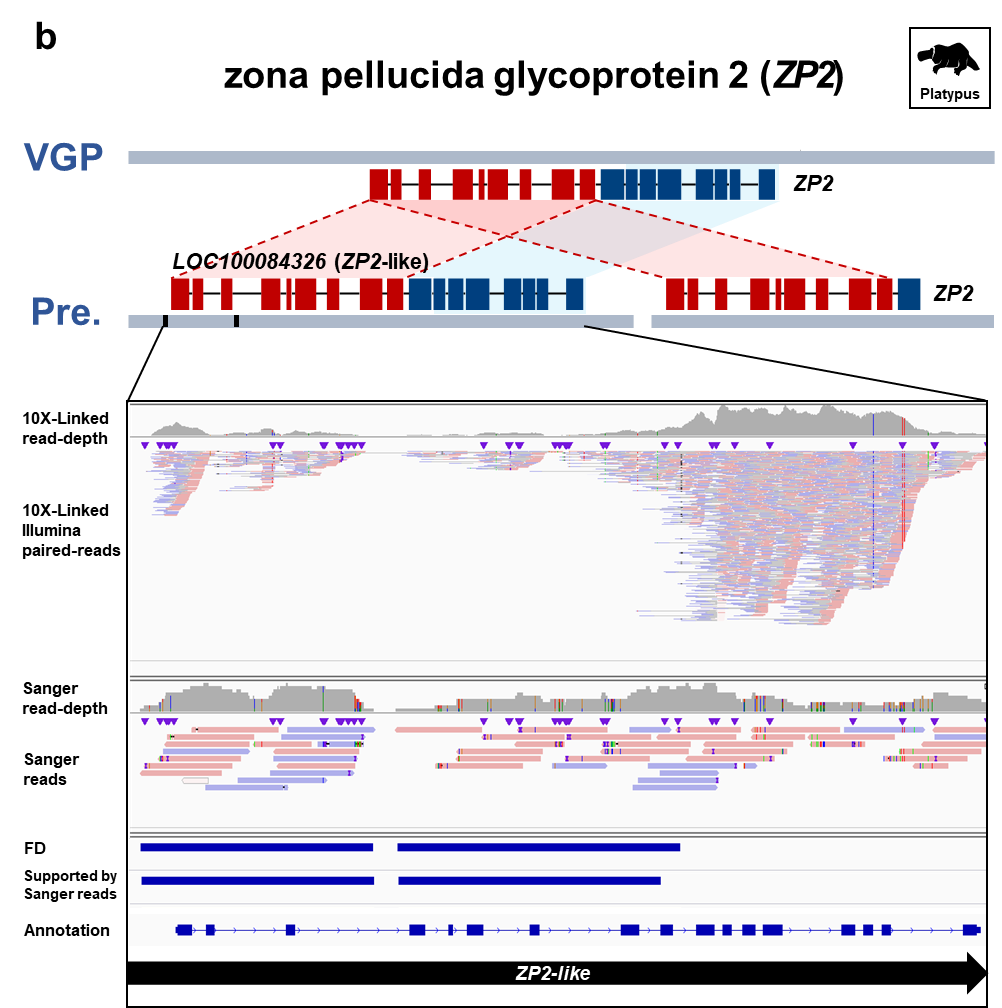


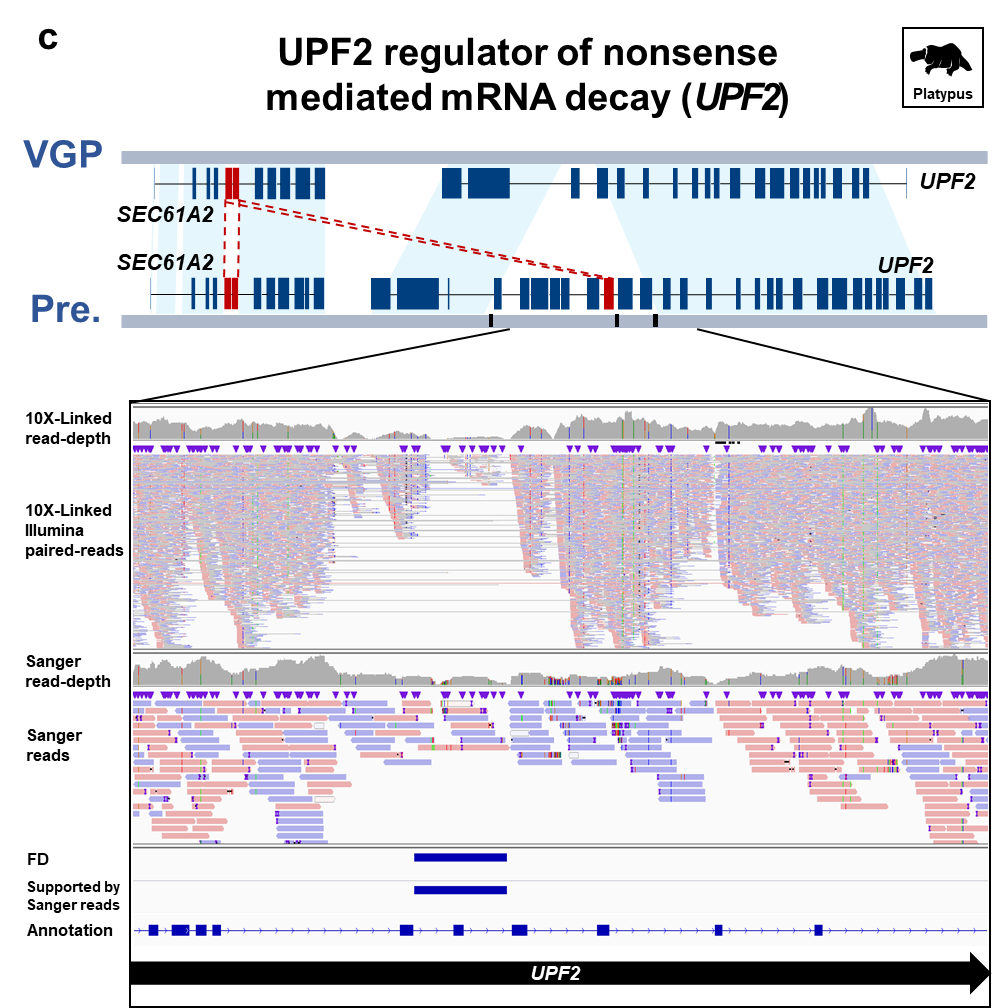


**Fig. S6 Genome landscape of platypus assembly false duplications using Sanger reads. a,** *HSF2* false duplication**, b,** *ZP2*-like false duplication. **c,** *UPF2* false duplication. 10X linked reads are shown as paired read alignments above the Sanger read alignments, along with the depth coverage of the respective read data. **‘**FD’ is the false duplication identified without Sanger reads. The region of false duplication that was supported by Sanger reads with under haploid level low read coverage is represented below ‘FD’.


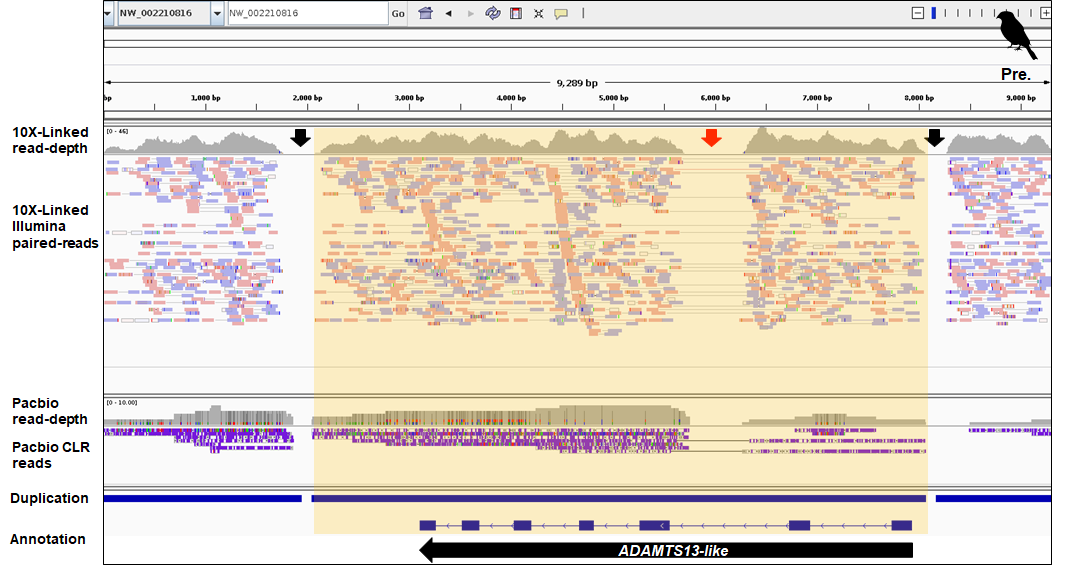


**Fig. S7 Additional findings for the genome false duplication landscape of the *ADAMTS13*-like gene.** This segment of the *ADAMTS13* gene is located in an unplaced scaffold of the previous zebra finch assembly. 10X linked reads are shown as paired read alignments above the PacBio CLR read alignments, along with the depth coverage of the respective read data. Black and red arrows represent an assembly gap and depth-gap, respectively.


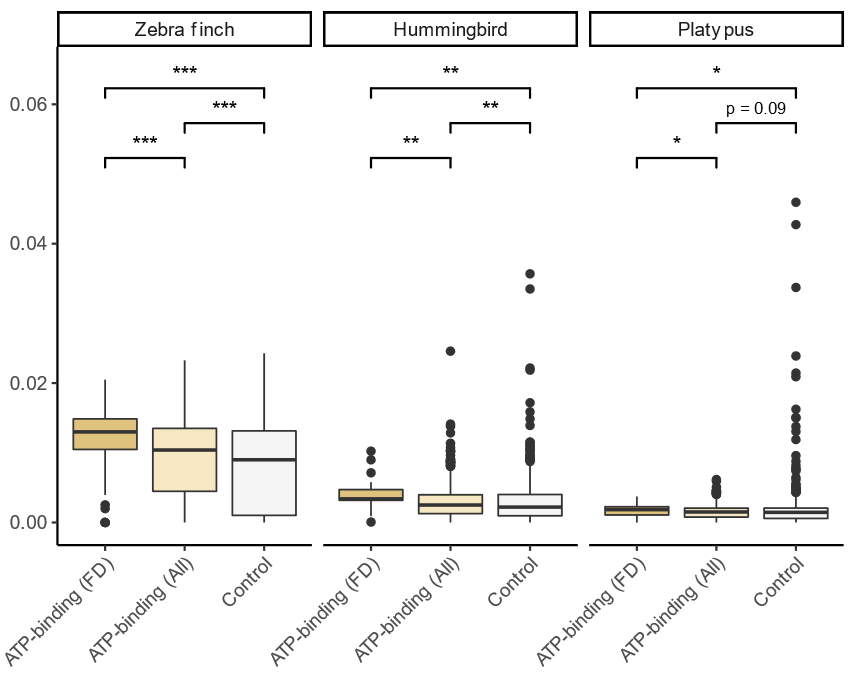


**Fig. S8 Heterozygosity of ATP-binding genes with or without false duplications.** ‘Control’ genes were randomly chosen for each species without ATP-binding genes. Box plots show median, first and third quartiles, range, and outliers as dots. One-sided Wilcoxon rank sum test was used to calculate significance between heterozygosity levels (******P*** < 0.001, *****P*** < 0.01, ****P*** < 0.05; one-sided).

**
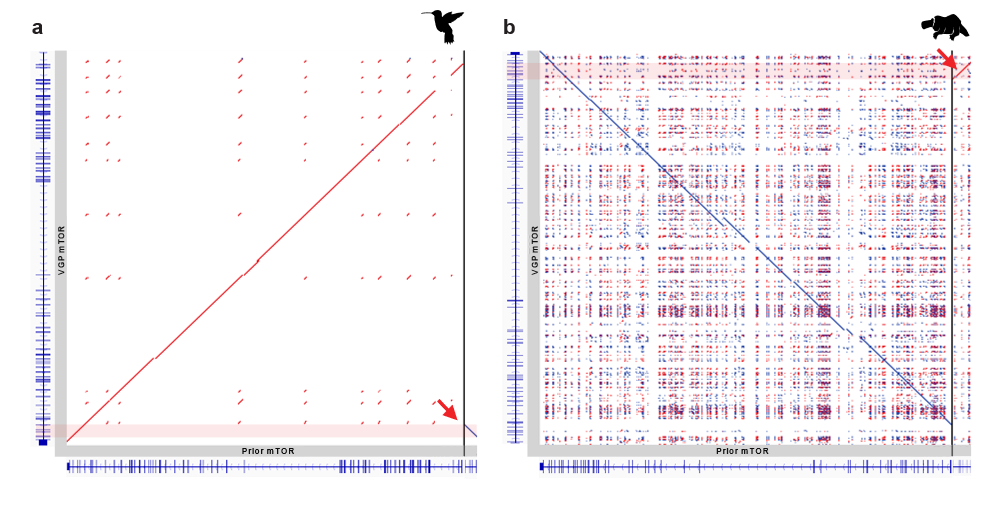
**

**Fig. S9 False duplications of the *MTOR* gene in the prior hummingbird (a) and the platypus (b) assemblies.** Alignment dot-plot shows *MTOR* gene alignment between the previous and the VGP assemblies. The alignment of the *MTOR*-like gene in each previous assembly is marked by a red arrow. The blue bars represent the exons of *MTOR* and *MTOR*-like genes. The platypus *MTOR* region is more repetitive than in the other species.


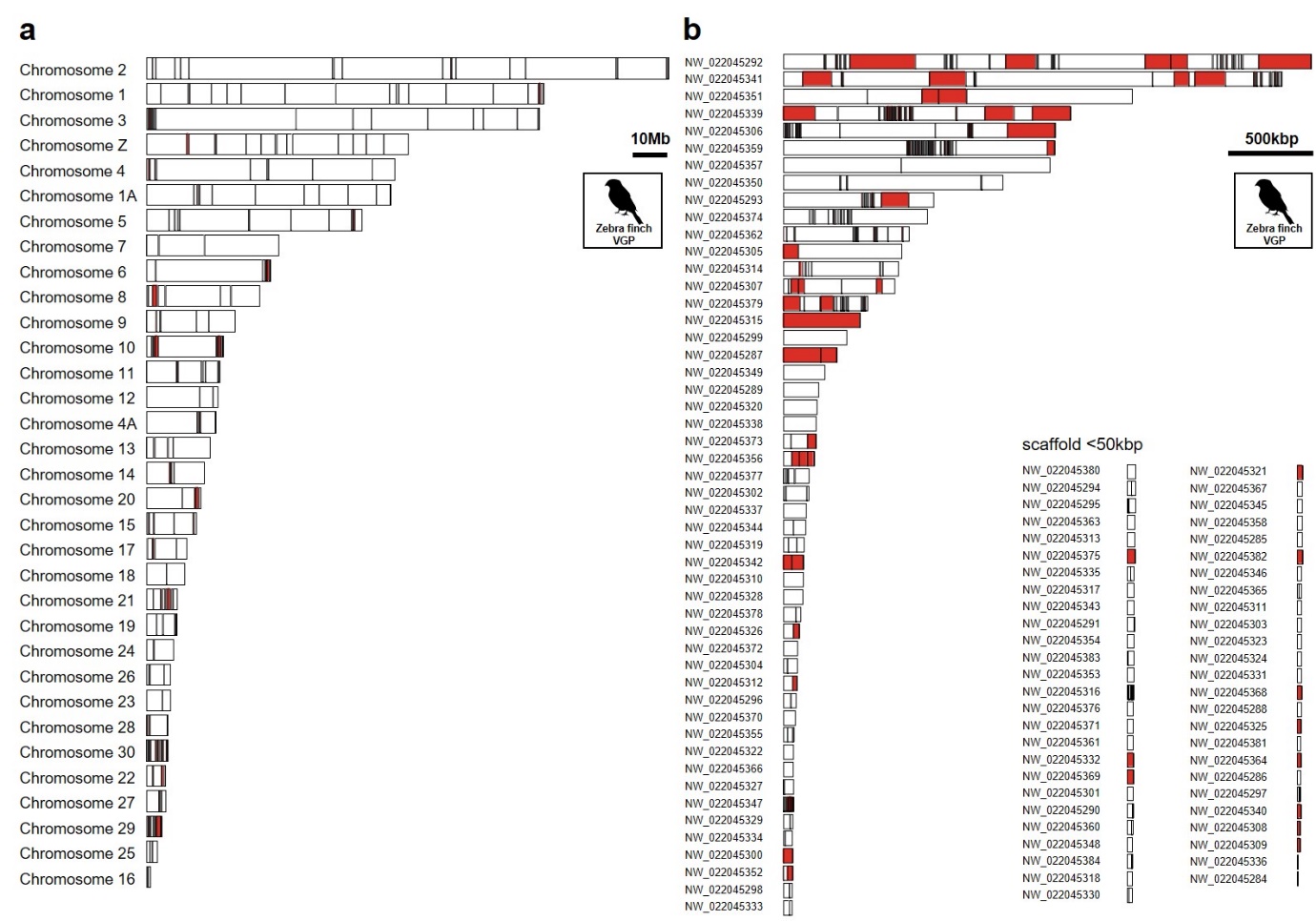


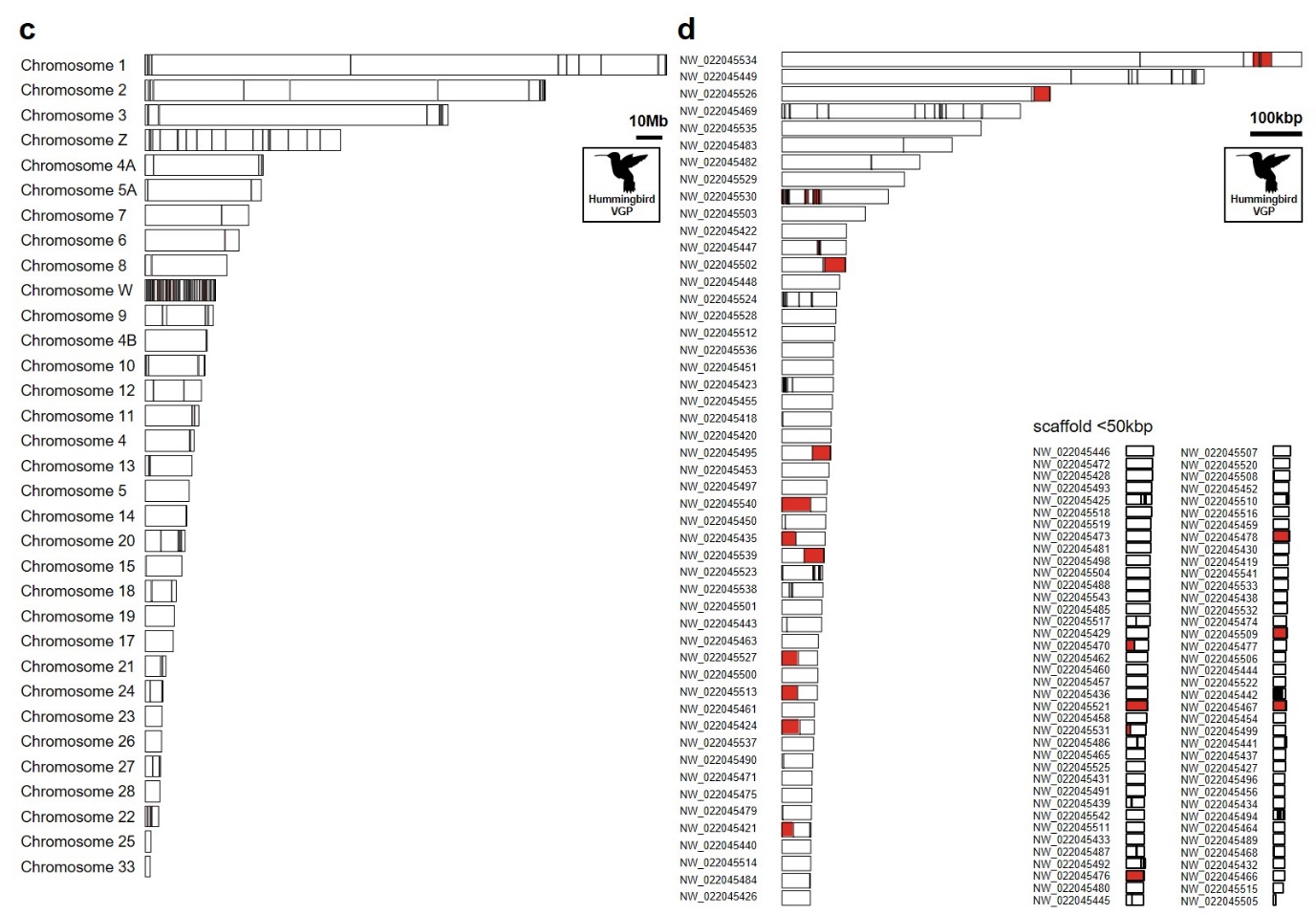


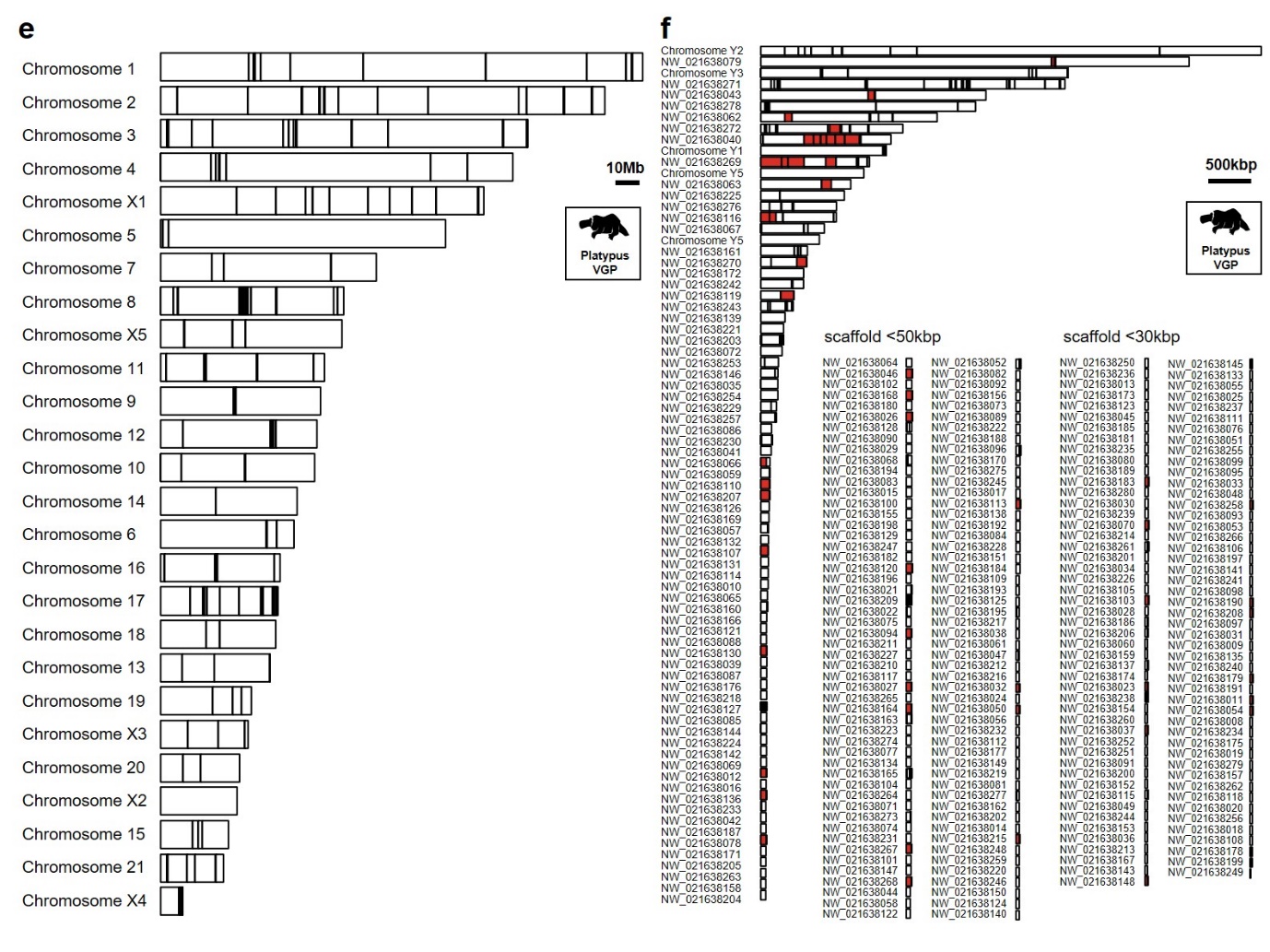


**Fig. S10 Chromosomal location of false duplications in the VGP assemblies.** False duplications are marked as small (black, <1kbp) or large (≥ 1kbp, red) bars, in each named chromosome (**a, c, e**) or unplaced scaffold (**b, c, d**) for the zebra finch (**a, b**), hummingbird (**c, d**) and platypus (**e, f**).

**
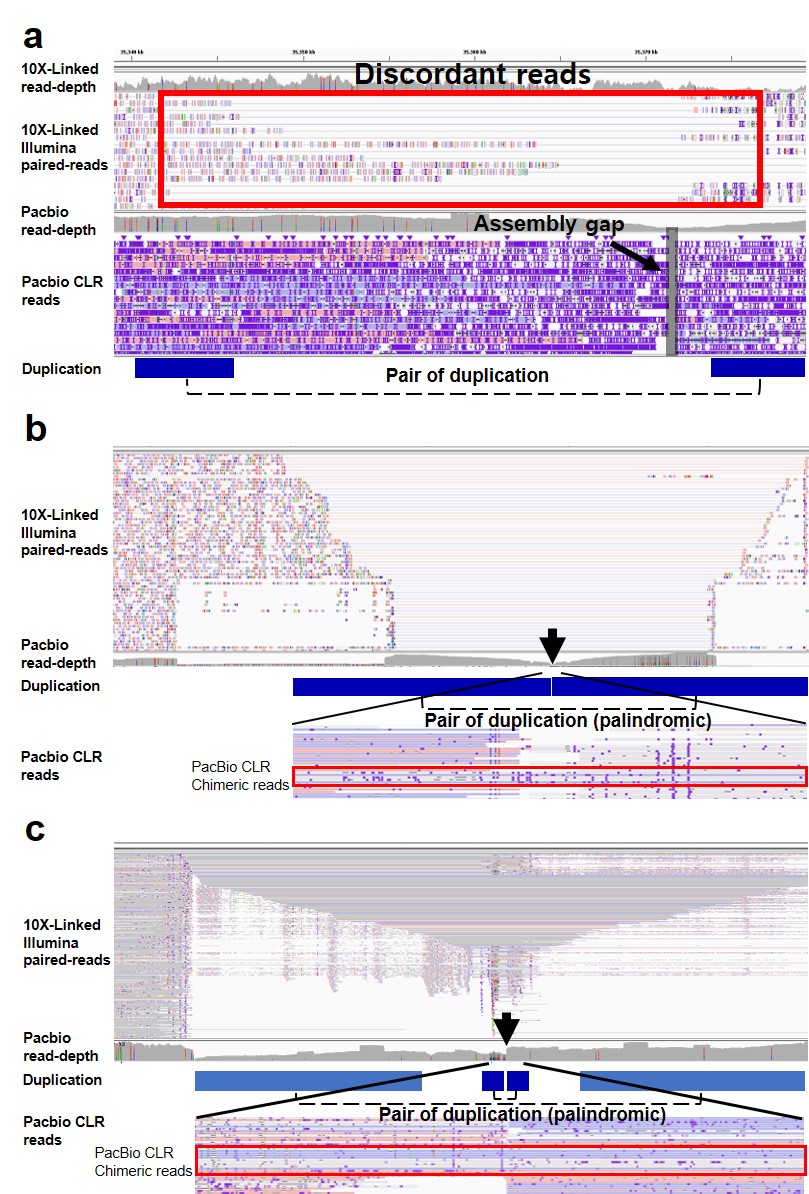
**

**Fig. S11 Example cases of false duplications in the VGP assemblies. a,** A false duplicated region on zebra finch chromosome 6, ~7 kbp long, with an assembly gap and discordant 10X linked reads around the duplication (red box). 10X linked reads are shown as paired read alignments above the PacBio CLR read alignments, along with the depth coverage of the respective read data. **b,** False duplication in zebra finch scaffold NW_022045321 caused by a PacBio sequence read chimera. The ~10 kbp of palindromic sequence was duplicated without an assembly gap. But this region includes a sequence depth-gap with 10X linked reads (black arrow), signifying sequencing artifacts connecting the two regions. The symmetric reduction of insert sizes of 10X linked reads signifies the duplication of palindromic sequence. Near the center of the duplication, the six PacBio reads containing the chimeric sequences overlapped 10X depth-gap connecting the two duplicated sequences (red box). **c,** A duplicated region on chromosome 17 of the platypus, similar to the chimeric type in (**b**) except the PacBio read depth has a more pyramid structure in the palindromic duplicated regions.


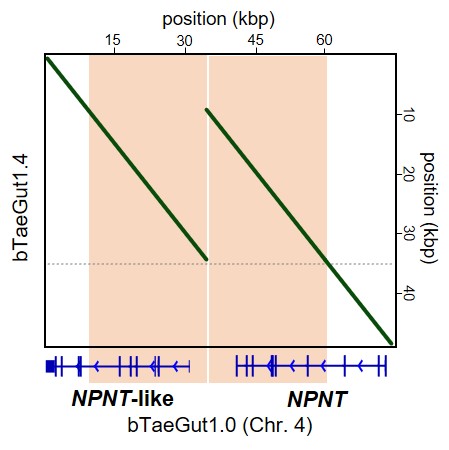


**Fig. S12 Correction of the *NPNT* gene in VGP v1.7 pipeline assembly.** Alignment dot-plot shows that the region with six duplicated exons of the *NPNT* gene in VGP v1.0 pipeline assembly (bTaeGut1.0) was prevented in the VGP v1.7 pipeline assembly (bTaeGut1.4). The blue bars represent exons of *NPNT* and *NPNT*-like genes in bTaeGut1.0.


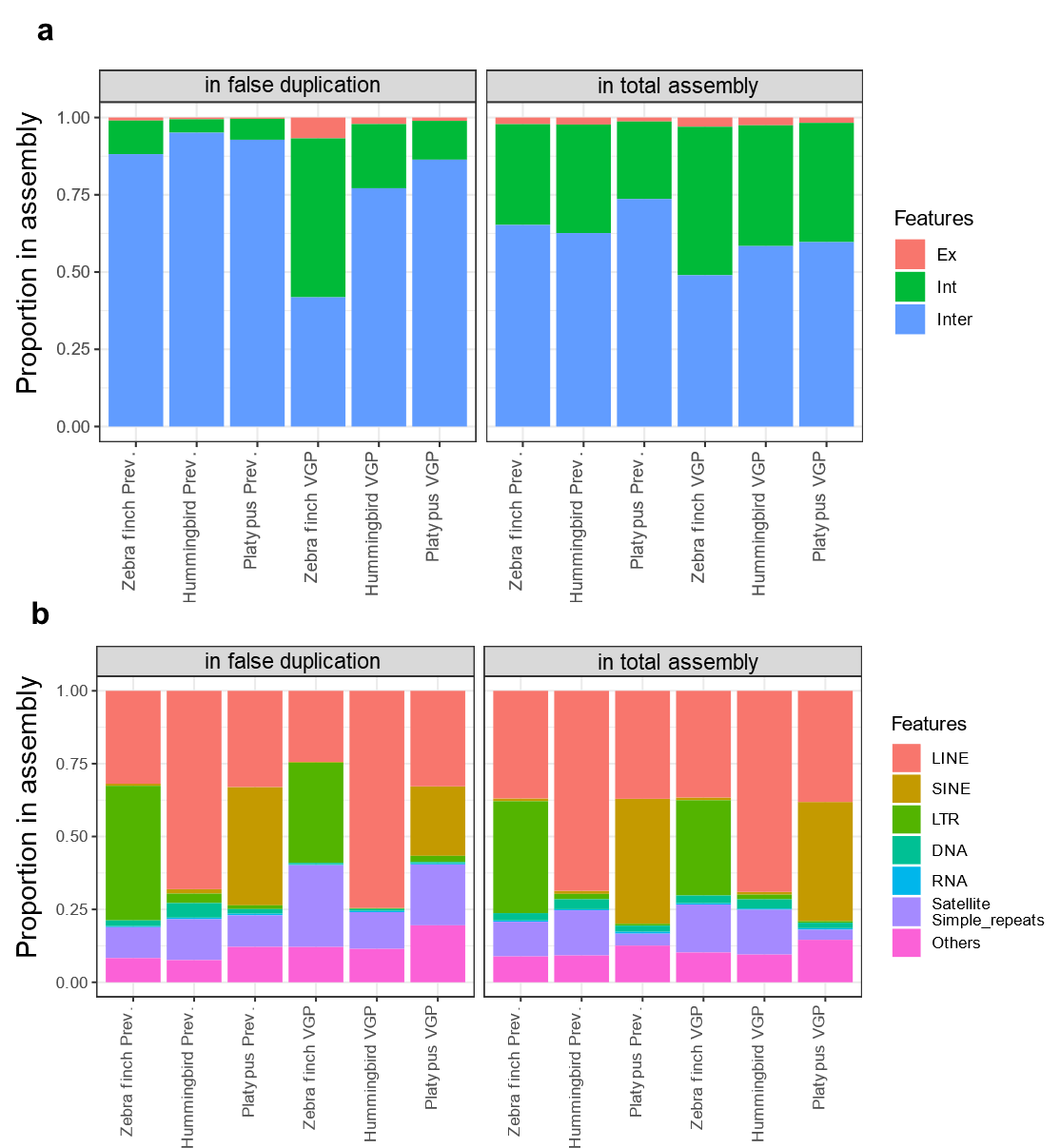


**Fig. S13 Proportions of genomic partitions represented among the falsely duplicated regions. a,** Proportion of false duplications among exon (Ex), intron (Int), intergenic (Inter) regions. **b,** Proportion of false duplications among different types of repetitive elements. Left panels show proportions among the false duplicated sequences; Right panels show proportions of all sequence types relative to the whole genome size.


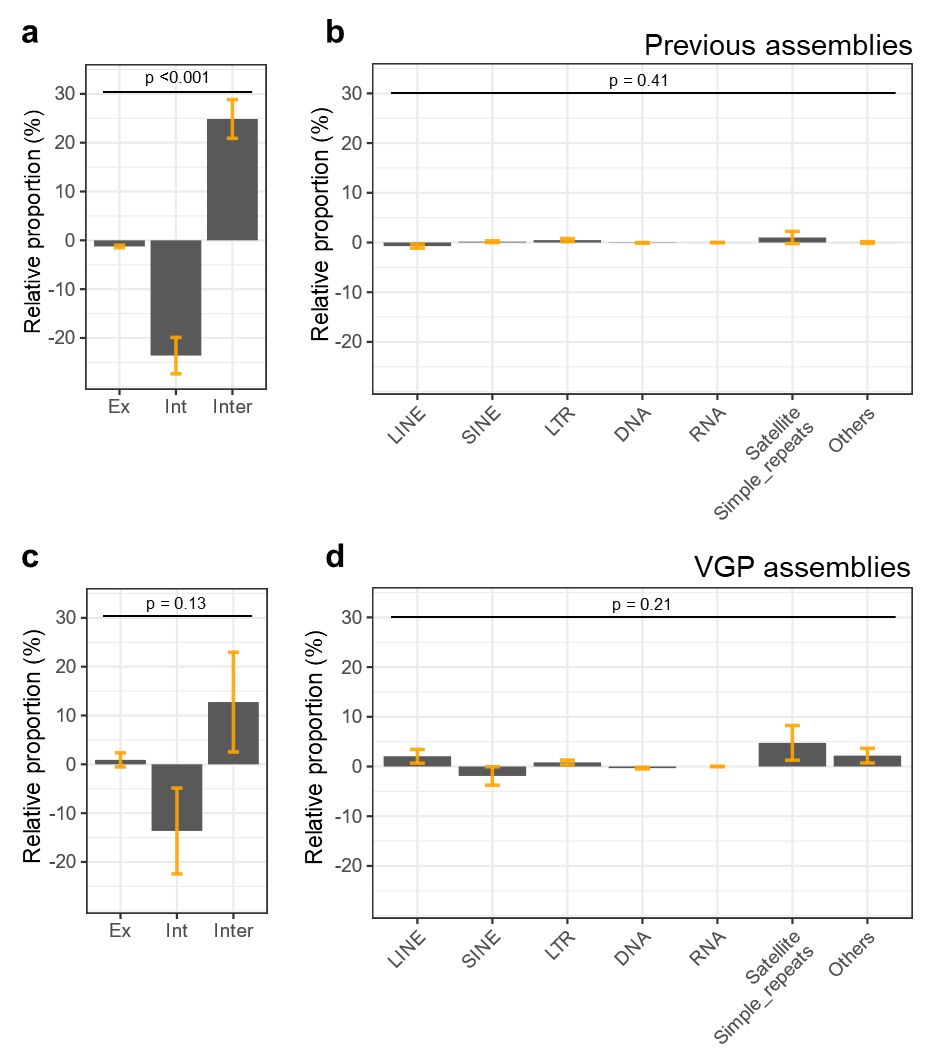


**Fig. S14 Difference of proportion of each genomic partition containing false duplications relative to expected frequency. a and** **b,** Relative proportions of false duplications in exon, intron, intergenic, and repeat regions in the previous assemblies. **c and d**, Relative values in the VGP assemblies. Error bars are standard error (*n* = 3 species each). The difference of relative proportion between genomic partitions was tested by one-way analysis of variance (ANOVA).


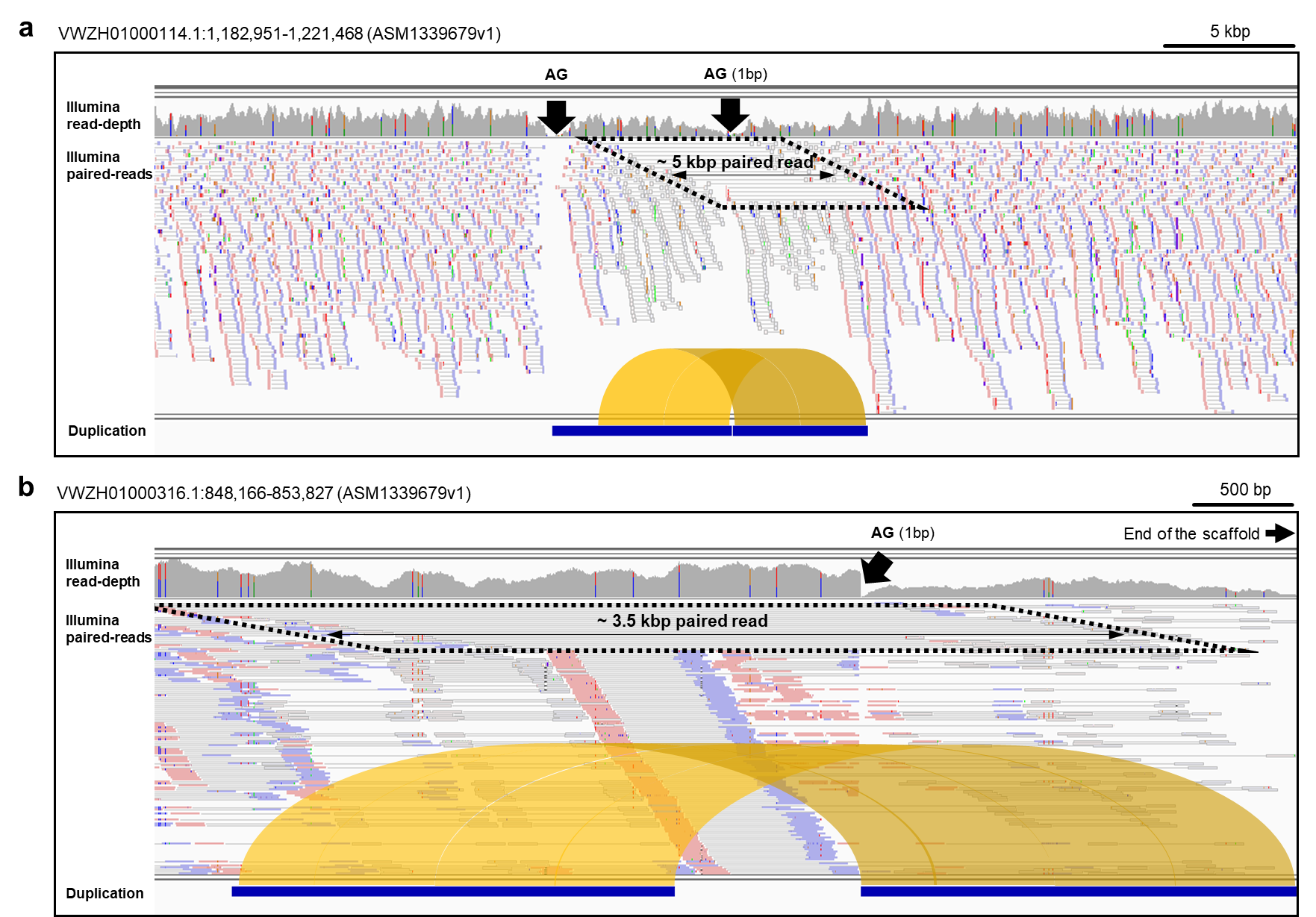


**
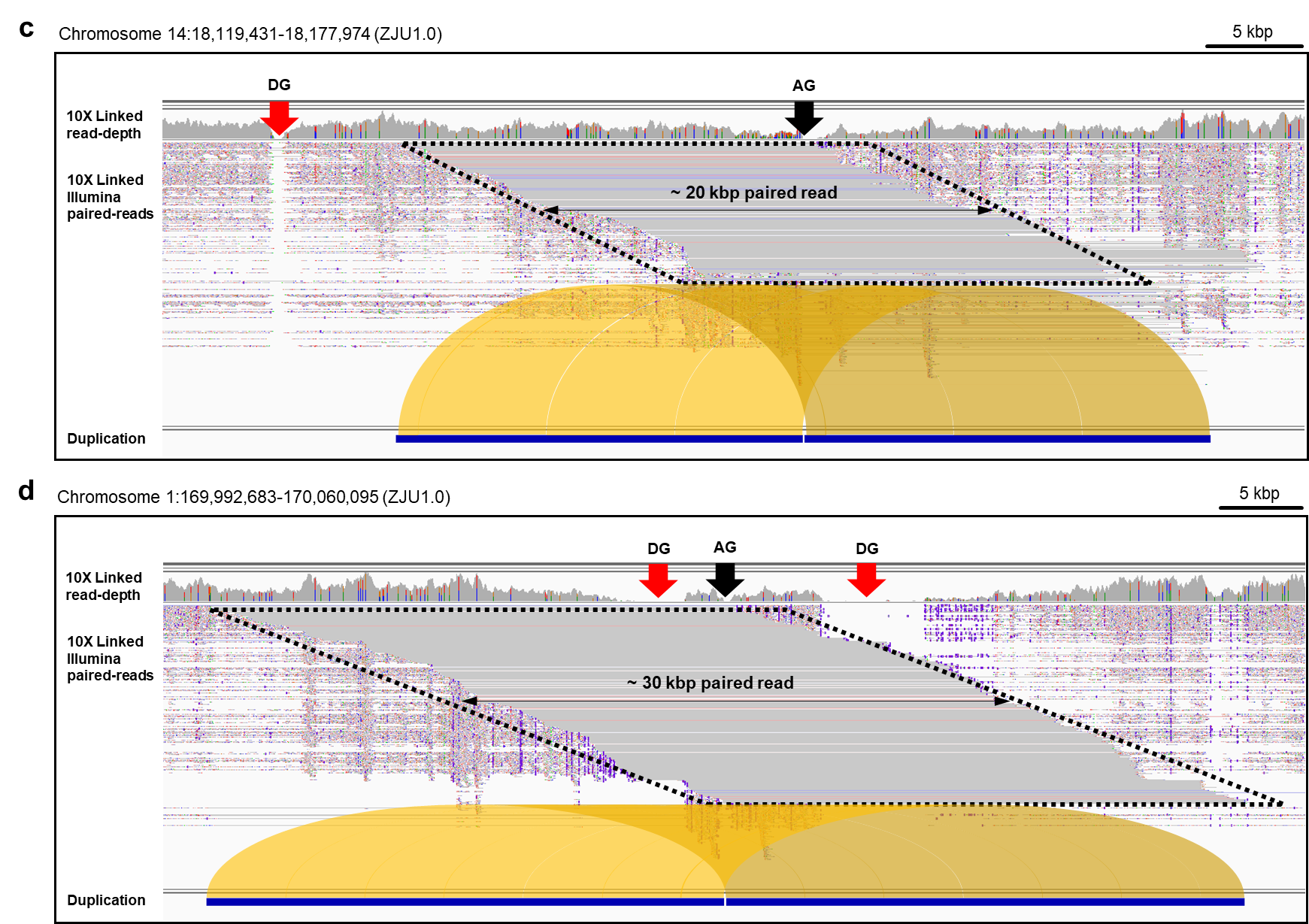
**

**Fig. S15 The genome landscape of false duplications in emu assemblies. a and b,** Duplicated region in the previous short-read based assembly. **c and d,** Duplicated region in the recent long-read based assembly. 10X linked reads and Illumina reads are shown as paired read alignments above the duplication region, along with the depth coverage of the respective read data. AG, assembly gap. DG, depth-gap.


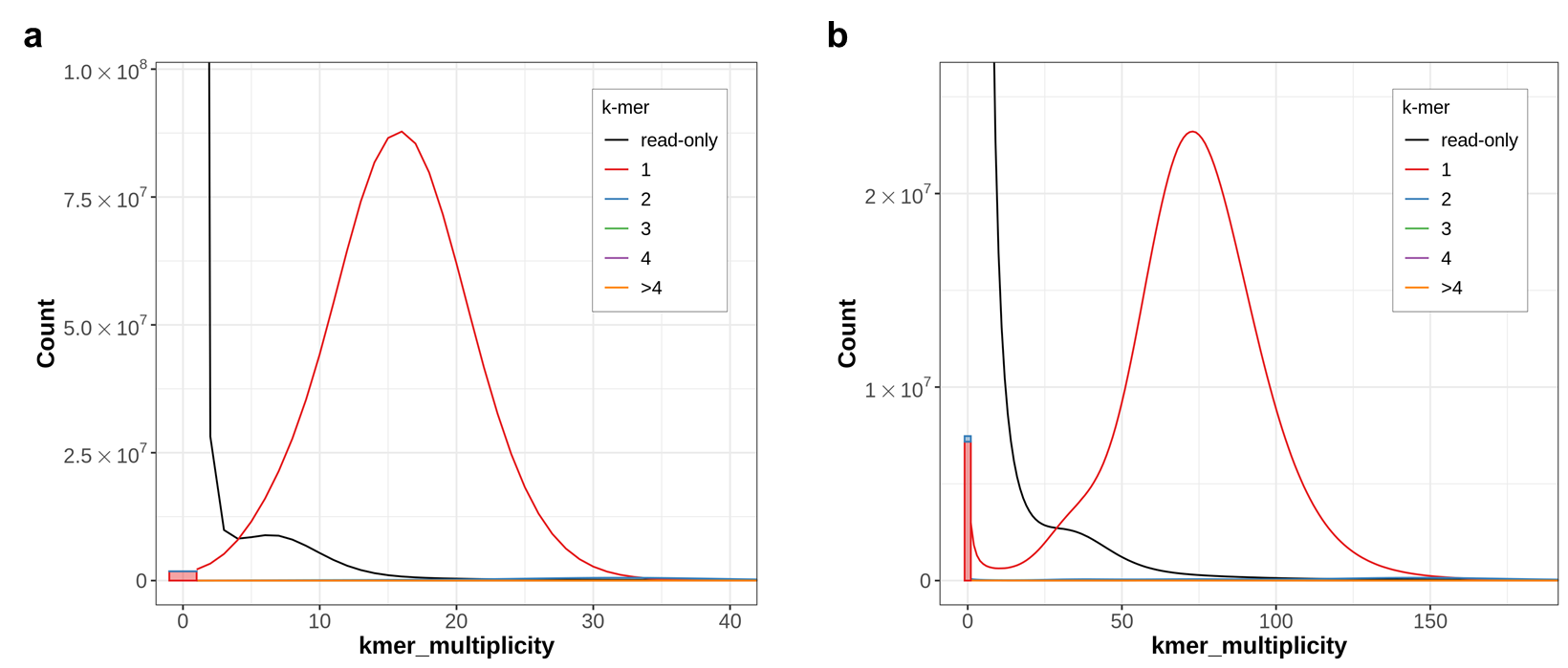


**Fig. S16 *K-mer* profiling for emu assemblies. a,** Previous short-read based assembly. **b,** Recent long-read based assembly. From the sequences of reads (Illumina shotgun for previous, 10X linked read for recent) and assemblies, *k-mer* multiplicity was calculated. The x-axis is the *k-mer* multiplicity calculated from the reads, and the numbers in the box represent the *k-mer* multiplicity found in the primary pseudo-haplotype assembly. *K-mer* multiplicity of 2 copies or higher under the area of single copies (red) are overly represented as false duplications.
