# Supplementary material for: Widespread false gene gains caused by duplication errors in genome assemblies
Source: Genome Biol. 2022 Sep 27;23:205. doi: 10.1186/s13059-022-02764-1 (PMC9516828; doi:10.1186/s13059-022-02764-1)
Supplement: Supplementary file 9 — Additional File 9. Review history. [file 13059_2022_2764_MOESM9_ESM.docx]

**1^st^ round**

**Reviewer 1**

This study considers the false duplications in three pairs of genome assemblies. In particular, they consider three species: platypus, zebra finch, Anna's hummingbird and compare the genome assembly in NCBI with the "newer" genome assembly produced recently by the Vertebrate Genome Project (VGP).

1) The first major question that I have is about validation of the assemblies. (a) First, as it is described on page 5 of the manuscript, my understanding was that biological samples taken from Anna's hummingbird and zebra finch were the exact same samples / individuals sequenced for both assemblies (VGP and older, publicly available assemblies) but platypus is not? If this is correct then I would argue that it is unclear whether the mis-assemblies are truly mis-assemblies or variation between the individuals of the same species? (b) Second, most of the validation seems to come for computational methods / k-mer counting and alignment and there's no PCR validation of these mis-assemblies? If this is correct, this makes me mildly uncomfortable since computational validation has been routinely shown to be error prone.

2) I will be honest and say that I struggled to determine what the take away is of this manuscript. The figures are nice and high quality, however, the paper reads more like a technical report as it is just reporting technical aspects of the three different assemblies and the study is unable to be reproduced in another species. The VGP data (long read, 10X sequencing / BioNano data) is unlikely to be produced by the average biological lab, and there is a vast, vast difference between this data and the data produced for the initial assemblies (2008 Sanger sequencing for example?). The data is no longer available for the first assemblies (if I read this correctly) so it's unclear whether the differences are due to advances in the computational work or the extensive data produced. I would prefer the reviewers choose an additional species where there at least similar data publicly available and run the same (or from the era) computational methods. The authors of this manuscript can equivalently take the long read data (maybe eliminate the 10X sequencing and bionano data) and assemble. This would at least help decipher if these mis-assemblies are due variation in the extensive data produced or advancements in the computational method.

Moreover, the results in the manuscript were unremarkable given how old the data and assemblies are. There was some discussion of there being an attempt to validate these assemblies but this needs to be discussed further in order to put the results in more context. Actually, when I read it I was more impressed with how decent the assembly. For example, figure 1(a) doesn't make the current assembly look so terrible.

3) Similar to my prior comment, it would have been beneficial to contextualize the results in light of the current research on these species. Given the genes that are affected and the prior work, is there any issues / related work that needs revising or revisiting? A lot of the errors were simply reported without any biological context.

3) A lot of the language in the manuscript is very specific to VGP. For example, "purge_dups" / "VGP v1.6 pipeline" / "VGP v1.7 pipeline" Moreover, a lot of citations are from VGP or labs related to VGP. I would suggest rewriting certain sections so it is appealing to a wider audience.

**Reviewer 2**

In the manuscript entitled "Widespread false gene gains caused by duplication errors in genome assemblies", Ko et al. explore how false duplications are generated, can be identified and classified in the genomes of platypus, zebra finch and Anna's hummingbird are cases of study. The authors conclude that most of the false duplications are mainly assembly errors due to heterozygous regions that are wrongly assembled as paralogs in haploid assemblies. One option to reduce this source of error is inbreeding, but it is frequently difficult to carry out. However, bioinformatics methods, like the ones presented in the manuscript, can help to remove false duplications in assemblies

The manuscript shows solid evidence about the assembly errors that can lead to a false duplication, and several practical procedures to detect and correct them. In general, I find the manuscript relevant for a broad audience. My main suggestion for the authors is if it would be possible to predict which genomic regions can be more prone to false duplications. Repetitive elements, like transposable elements or satellite DNA interfere in assemblies, but I wonder if authors can discuss a bit more about the genomic characteristics that provoke false duplications.

In addition to this, I have few minor comments.

lines 96-97: "The VGP version of the assemblies were long-read based. We found widespread false duplications". I think this sentence sounds a bit isolated in the text and suggests to integrate it in the beginning of the paragraph using the proper connectors.

lines 103-121: I consider that this paragraph fits better in the Background section, since it is a description of the previous assemblies.

lines 224-229: Figure 3. It was a bit hard for me to realize that the mappings in the top are the ones from the 10xGenomics data and the mappings in the bottom are the ones from the PacBio data. Please, clarify this in the legend of this figure and figures 5, S3, S4 and S7.

lines 339-342: Figure 6. I miss in the legend some information about the color (red to black) from the right subpanels.

lines 457-459: Please indicate the GitHub link for the purge_dups pipeline.

**Reviewer 3**

This work studied the problem of false duplications in current genome assemblies and its effects on downstream analysis pipelines (gene annotation, etc). The authors first identified candidate duplications from sequence similarity using whole genome alignment (to itself and to previous version of the assembly). The candidates were further finalized using purge_dups. The false duplications are further classified to heterotype and homotype duplications based on k-mers profiles.

This paper raised a common type of misassemblies (false duplications) that evidently exists in most genome assemblers. While authors showed a list of steps and pipelines tools (all parameters are chosen without any specific justification) - they don't have contribution in algorithmic work of identifying false duplication. No specific examples were shown why such misassemblies were generated in any of the assemblers' and scaffoldings' algorithms.

I believe the topic that the authors choose to work is important, and the community would benefit from this analysis if it could be done on at least on a significant of number of assemblies generated using various assemblers ( 3 assemblies are too small to conclude about the error (false duplication) landscape of current genome assemblies).

**Authors’ response**

Reviewer #1:
Reviewer #1 comment: This study considers the false duplications in three pairs of genome assemblies. In particular, they consider three species: platypus, zebra finch, Anna's hummingbird and compare the genome assembly in NCBI with the "newer" genome assembly produced recently by the Vertebrate Genome Project (VGP).
1) The first major question that I have is about validation of the assemblies. (a) First, as it is described on page 5 of the manuscript, my understanding was that biological samples taken from Anna's hummingbird and zebra finch were the exact same samples / individuals sequenced for both assemblies (VGP and older, publicly available assemblies) but platypus is not? If this is correct then I would argue that it is unclear whether the mis-assemblies are truly mis-assemblies or variation between the individuals of the same species? (b) Second, most of the validation seems to come for computational methods / k-mer counting and alignment and there's no PCR validation of these mis-assemblies? If this is correct, this makes me mildly uncomfortable since computational validation has been routinely shown to be error prone.
Response: We appreciate the reviewer’s comments and suggestions. (a) For the first concern, yes, the Anna’s hummingbird and zebra finch prior and new VGP assemblies were generated from the same individuals, and thus the differences can’t be because of individual differences. The platypus' prior and new VGP assemblies are from different individuals. The results of the platypus were similar to the findings with hummingbird and zebra finch assemblies, and consistent with assembly errors as opposed to different individuals. To address the reviewer’s concern, we added some explanation and performed additional analyses on the platypus. First we note that it is unlikely that a chromosomes worth of DNA (126 Mbp) or 6% of the genome, comprising 736 genes scattered on many chromosomes around was naturally gained or lost by duplication or deletion, respectively, in one individual versus another of the same species and same population. Second, to address the reviewer’s concern experimentally, we found the raw Sanger sequence read data used for the previous platypus assembly in the NCBI trace archive. Although only 8X depth-coverage, we mapped that read data back to the prior assembly, and were able to demonstrate when possible through half depth coverage that many of the previous gene duplications were in fact alternative haplotypes and thus false duplications, and thus not individual differences between animals. This new result is now added in the results section titled ‘False duplications in previous and VGP assemblies’ and ‘False duplications cause false annotation errors’ with Fig. S6.
(b) In terms of computational versus experimental validation by PCR, we do not have access to the platypus sample used to create the prior genome assembly over 13 years ago. However, we have sufficient amounts of raw single molecule Pacbio-reads, which is also sufficiently longer (10-30 kb) than Sanger sequence reads of PCR products (0.5-0.7 kb). We find that the majority of the false duplications are near each other and have a gap between them, and when corrected in the new assemblies have single molecule raw reads that span the gaps of the prior assembly but with no duplicated gene, further validating with raw data that they are false duplications in the prior assembly, and not computational errors. This result is also now included in the revised manuscript.

Reviewer #1 comment: 2) I will be honest and say that I struggled to determine what the take away is of this manuscript. The figures are nice and high quality, however, the paper reads more like a technical report as it is just reporting technical aspects of the three different assemblies and the study is unable to be reproduced in another species. The VGP data (long read, 10X sequencing / BioNano data) is unlikely to be produced by the average biological lab, and there is a vast, vast difference between this data and the data produced for the initial assemblies (2008 Sanger sequencing for example?). The data is no longer available for the first assemblies (if I read this correctly) so it's unclear whether the differences are due to advances in the computational work or the extensive data produced. I would prefer the reviewers choose an additional species where there at least similar data publicly available and run the same (or from the era) computational methods. The authors of this manuscript can equivalently take the long read data (maybe eliminate the 10X sequencing and bionano data) and assemble. This would at least help decipher if these mis-assemblies are due variation in the extensive data produced or advancements in the computational method.
Moreover, the results in the manuscript were unremarkable given how old the data and assemblies are. There was some discussion of there being an attempt to validate these assemblies but this needs to be discussed further in order to put the results in more context. Actually, when I read it I was more impressed with how decent the assembly. For example, figure 1(a) doesn't make the current assembly look so terrible.

Response: The main take-away message of this study is that there are thousands of false gene and genic region duplications in prior and current assemblies that do not take haplotype differences or sequence errors into account, and that these differences are higher the greater the divergence between haplotypes in given regions of the genome or even within a single gene. Most of the false duplications are generated because of the computational method, not appropriately phasing haplotypes during genome assembly. However, the new long-read data does improve the ability to phase haplotypes for longer stretches of sequence. Pacbio sequencing and other new technologies are becoming increasingly available. As of to date, there are 255 vertebrate genome assemblies in NCBI that have used a Pacbio platform and achieved the VGP N50 metrics. Of these 151 were generated by the VGP group (NCBI BioProject PRJNA489243), and the remaining ~100 by others. However, not all of those other assemblies were properly haplotype phased and thus many have false duplication errors. One of the additional assemblies we analyzed for this studys, the emu, had 0.1% false duplications for the prior short read and 1.1% false duplications for the new long reads assembly; we are now working with the generators of the recent genome to correct and to include it with the VGP list. This message has to get out, and we believe our study will help do so.
For elimination of different data types and the impact on false duplications and other assembly errors, this was already done in our flagship companion paper by Rhie et al (2021) in Nature, which we now state in the manuscript. But Rhie et al. (2021) didn’t evaluate how many false duplications in the assemblies are made by various strategies. So, in response to the reviewer’s comment, we measured the amount of false duplications for the different hummingbird assembly benchmarks, and their causes. We confirm that the combination of having long reads and purging false duplications before scaffolding has a greater impact on reducing false duplications overall. We have now revised the manuscript with these additional analyses and made the above items clearer, including the take-away message.
For Figure 1a, we are not sure if the reviewer means the VGP or prior assembly as ‘current’? If the reviewer means the VGP assemblies doesn’t look so terrible, it is because they are much improved compared to the prior assemblies. If the reviewer is referring to the prior assembly as current in the Figure 1a right most panel, the prior assembly has 3 out of 4 false duplicated genes, with the VGP assembly having 0 in this genic region model. We think that is a dramatic difference. We further emphasized this difference in the figure legend.

Reviewer #1 comment: 3) Similar to my prior comment, it would have been beneficial to contextualize the results in light of the current research on these species. Given the genes that are affected and the prior work, is there any issues / related work that needs revising or revisiting? A lot of the errors were simply reported without any biological context.
Response: We are glad the reviewer brought this up. Yes, there are issues of biological interpretation of the prior assemblies and prior and current literature with false duplications. We had examples, and now included additional published examples following the reviewer’s request. For example, we found that reported ADAMTS13 and neurotrypsin gene duplications in the zebra finch (Quesada et al., 2010; Warren et al., 2010) are in fact false duplications (added to Fig. 5). These additional findings are presented in a new paragraph in the section ‘False duplications cause false annotation errors’.
The reviewer’s comment on biological context (and a comment from Reviewer 2 on bias of false duplications to certain genes) also prompted us to consider whether certain types of genes are more likely to be falsely duplicated over others, perhaps because of higher divergence rates between haplotypes. To test this idea, we first performed gene ontology enrichment based on the list of genes with false duplication. We found that genes with GO terms for nucleotide binding (e.g. ‘ATP-binding’) were enriched for false duplications in all three species (new Fig. 6). These included the MTOR gene, which regulates growth, metabolism, signaling, and disease with the kinase domain using ATP (Fig. 7a,b; Additional file 1: Fig. S9), and ‘ABC transporter’ genes in zebra finch and platypus (Additional file 6: Table S5), These ABC transporter genes are one of the largest and oldest gene superfamilies in diverse organisms from prokaryotes to vertebrates, and plays key roles in encoding membrane proteins that transport diverse metabolites. The heterozygosity levels of these genes were significantly higher than the average (Additional file 1: Fig. S8). These new findings have been added to the manuscript.

Reviewer #1 comment: 3) A lot of the language in the manuscript is very specific to VGP. For example, "purge_dups" / "VGP v1.6 pipeline" / "VGP v1.7 pipeline" Moreover, a lot of citations are from VGP or labs related to VGP. I would suggest rewriting certain sections so it is appealing to a wider audience.

Response: We tried to reduce abbreviations, and spelled out in more detail the meaning of the different terms to make them more accessible to a general audience. The VGP has most of the world’s leading genome experts participating in the project, involving hundreds of labs, with the goal of generating high quality assemblies of all 70,000 vertebrate species. Thus it is hard to find a recent paper that generates such high-quality assemblies that is not in some form associated with the VGP. Nevertheless, we appreciate the reviewer’s comment towards fairness of citations, and included some additional citations of persons who have as of yet have not collaborated with the VGP.

Reviewer #2:
Reviewer #2 comment: In the manuscript entitled "Widespread false gene gains caused by duplication errors in genome assemblies", Ko et al. explore how false duplications are generated, can be identified and classified in the genomes of platypus, zebra finch and Anna's hummingbird are cases of study. The authors conclude that most of the false duplications are mainly assembly errors due to heterozygous regions that are wrongly assembled as paralogs in haploid assemblies. One option to reduce this source of error is inbreeding, but it is frequently difficult to carry out. However, bioinformatics methods, like the ones presented in the manuscript, can help to remove false duplications in assemblies The manuscript shows solid evidence about the assembly errors that can lead to a false duplication, and several practical procedures to detect and correct them. In general, I find the manuscript relevant for a broad audience. My main suggestion for the authors is if it would be possible to predict which genomic regions can be more prone to false duplications. Repetitive elements, like transposable elements or satellite DNA interfere in assemblies, but I wonder if authors can discuss a bit more about the genomic characteristics that provoke false duplications.
Response: We are glad to see the reviewer’s positive feedback on this paper. Following the reviewer’s request, we performed additional analyses to further investigate if there were specific genomic partitions are more prone to false duplications. We found that in the short read based assemblies, intergenic regions were the most dominant genomic partition among false duplications, even after normalizing to relative size of the genome. This was followed by introns, LTRs by not LINE repeats, and exons being the least likely among false duplications, despite the thousands of exon false duplications we found. In the VGP long-read assemblies the overall pattern was similar, except that repeat sequence were the highest proportion among the false duplications. This difference is presumably related to the greater success of separating haplotypes in non-repetitive regions with long-reads in the VGP assemblies, leaving the need for greater improvement in repetitive regions. In addition, following up a comment made by reviewer 2, among the genes, nucleotide binding (e.g. MTOR and ABC transporters; new Fig. 7; Additional file 1: Fig. S8) had a greater probability of false duplications across species than other gene categories, due to higher divergence of this more ancient gene family compared to others. All of these new findings have been added to the manuscript.

In addition to this, I have few minor comments.

Reviewer #2 comment: (1) lines 96-97: "The VGP version of the assemblies were long- read based. We found widespread false duplications". I think this sentence sounds a bit isolated in the text and suggests to integrate it in the beginning of the paragraph using the proper connectors.
Response: We thank the reviewer for this suggestion. We merged these sentences with a proper connector and moved it in the beginning of the paragraph.

Reviewer #2 comment: (2) lines 103-121: I consider that this paragraph fits better in the Background section, since it is a description of the previous assemblies.
Response: We agree, but we also think this paragraph has an advantage in ‘Results’ for the readers to understand what is difference between the previous and VGP assemblies. Thus we have mentioned it in both places.

Reviewer #2 comment: (3) lines 224-229: Figure 3. It was a bit hard for me to realize that the mappings in the top are the ones from the 10xGenomics data and the mappings in the bottom are the ones from the PacBio data. Please, clarify this in the legend of this figure and figures 5, S3, S4 and S7.
Response: We revised the legends of Figures 3, 5, S3, S4 and S7 (now Figure S11) to improve readability for the 10x and Pacbio mapping data.

Reviewer #2 comment: (4) lines 339-342: Figure 6. I miss in the legend some information about the color (red to black) from the right subpanels.
Response: We added the color information in the legend of Figures 6 (now Figure 8).

Reviewer #2 comment: (5) lines 457-459: Please indicate the GitHub link for the purge_dups pipeline.
Response: We added the GitHub link for the updated purge_dups for our paper.

Reviewer #3:
Reviewer #3 comment: This work studied the problem of false duplications in current genome assemblies and its effects on downstream analysis pipelines (gene annotation, etc). The authors first identified candidate duplications from sequence similarity using whole genome alignment (to itself and to previous version of the assembly). The candidates were further finalized using purge_dups. The false duplications are further classified to heterotype and homotype duplications based on k- mers profiles.
This paper raised a common type of misassemblies (false duplications) that evidently exists in most genome assemblers. While authors showed a list of steps and pipelines tools (all parameters are chosen without any specific justification) - they don't have contribution in algorithmic work of identifying false duplication. No specific examples were shown why such misassemblies were generated in any of the assemblers' and scaffoldings' algorithms.
Response: We thank the reviewer for pointing out these concerns. We have added the explanation of the specific parameters we chose, in the materials and methods. Some of these were chosen by empirical analyses to determine which parameters most reliably identify false duplications. This will be useful to others that wish to identify and filter out false duplications from their assemblies
In terms of contribution to algorithmic work, the main purpose of this study is not to develop an algorithm to remove false duplications, but to quantify the level, type, and potential causes of false duplications in prior and the new VGP assemblies. We are expecting a lower amount in the VGP assemblies, because they used long reads and haplotype phasing, but the prior studies did not present a thorough quantification of the improvements. This did not require developing new algorithms, although it required developing new method and pipelines. We did make some changes to source codes, however. We started with open source codes from the VGP to identify false duplication and gene gain on a Github. But we found that the codes for this approach were not very accessible. So, we modified the source codes to improve accessibility and they are now also available in a Github associated with this study (https://github.com/KoByungJune/FalseDuplication). In terms of specific examples of why such misassemblies were generated, we showed several examples in the previous submission, and have increased the number of examples shown (Figures 5e-h, 7). These examples show that how assembly algorithms handle differences in regions of higher versus lower heterozygosity and sequence errors are the primary culprits of the false duplications. We have now more clearly indicated the culprits in each of the examples shown.

Reviewer #3 comment: I believe the topic that the authors choose to work is important, and the community would benefit from this analysis if it could be done on at least on a significant of number of assemblies generated using various assemblers ( 3 assemblies are too small to conclude about the error (false duplication) landscape of current genome assemblies).
Response: We are happy to see that the reviewer views our topic as important. In terms of working on more than 3 species (6 assemblies: prior and VGP), although we made the VGP pipeline more routine since we began this study, these 6 assemblies have been monumental efforts, and expensive. The VGP assemblies required many iterations to get them correct. The analyses here also required a great amount of effort and expense. Nevertheless, in response to the reviewer’s concern, we further checked the prevalence of false duplication in another species, emu, the one made by Illumina reads and the other made by Pacbio, 10X and Hi-C data. We found that these assemblies included 1 Mbp and 14 Mbp (0.1 and 1.1% of total length) of false duplication, respectively (new Fig. S15). We also discovered false gene gain of the MTOR gene, which is one of ATP-binding genes in the short-read based assemblies of white-throated tinamou and domestic water buffalo. These new results support a needed awareness and handling of false duplication for modern assemblies. The reason why the new assembly has greater false duplications is because the generators of that assembly did now use a false duplication purging algorithm. We are now doing so to improve the new emu assembly, to include it in the VGP. In terms of adding comparisons of many more species assemblies to this study, when we began the study there were only 16 VGP assemblies, of which only four had prior assemblies to compare with. As of to date, there are now 151 VGP assemblies, with a proportion of them with prior counterparts. We prefer to publish this first study with three of the founding VGP species and assemblies, and then after analyze currently unpublished assemblies to get a better understanding of statistics of false duplications across species and different assembly and sequence data types. This study provides a valuable guide for such future studies. This rationale is now mentioned in the revised discussion.

**2^nd^ round**

**Reviewer 1**

|  | All my comments have been adequately addressed.  About this comment: "Response: We tried to reduce abbreviations, and spelled out in more detail the meaning of the different terms to make them more accessible to a general audience. The VGP has most of the world’s leading genome experts participating in the project, involving hundreds of labs, with the goal of generating high quality assemblies of all 70,000 vertebrate species. Thus it is hard to find a recent paper that generates such high-quality assemblies that is not in some form associated with the VGP. Nevertheless, we appreciate the reviewer’s comment towards fairness of citations, and included some additional citations of persons who have as of yet have not collaborated with the VGP."  The authors are being a little bit hubristic. There are plenty of well-regarded researchers in assembly that are not part of the VGP project. The authors should ensure their research is representative not as just a "VGP project" but as a scientific one that has a broad perspective that is well-positioned in the current research. |
| --- | --- |
